# Quantification and mitigation of byproduct formation by low-glycerol-producing *Saccharomyces cerevisiae* strains containing Calvin-cycle enzymes

**DOI:** 10.1186/s13068-023-02329-9

**Published:** 2023-05-12

**Authors:** Aafke C. A. van Aalst, Mickel L. A. Jansen, Robert Mans, Jack T. Pronk

**Affiliations:** 1 Department of Biotechnology, Delft University of Technology, Van der Maasweg 9, 2629 HZ Delft, The Netherlands; 2 DSM Biotechnology Centre, Alexander Fleminglaan 1, 2613 AX Delft, The Netherlands

**Keywords:** Chemostat, Acetaldehyde, Acetate, Phosphoribulokinase, RuBisCO, Anaerobic, Ethanol, Redox

## Abstract

**Background:**

Anaerobic *Saccharomyces cerevisiae* cultures require glycerol formation to re-oxidize NADH formed in biosynthetic processes. Introduction of the Calvin-cycle enzymes phosphoribulokinase (PRK) and ribulose-1,5-bisphosphate carboxylase/oxygenase (RuBisCO) has been shown to couple re-oxidation of biosynthetic NADH to ethanol production and improve ethanol yield on sugar in fast-growing batch cultures. Since growth rates in industrial ethanol production processes are not constant, performance of engineered strains was studied in slow-growing cultures.

**Results:**

In slow-growing anaerobic chemostat cultures (*D* = 0.05 h^−1^), an engineered PRK/RuBisCO strain produced 80-fold more acetaldehyde and 30-fold more acetate than a reference strain. This observation suggested an imbalance between in vivo activities of PRK/RuBisCO and formation of NADH in biosynthesis. Lowering the copy number of the RuBisCO-encoding *cbbm* expression cassette from 15 to 2 reduced acetaldehyde and acetate production by 67% and 29%, respectively. Additional C-terminal fusion of a 19-amino-acid tag to PRK reduced its protein level by 13-fold while acetaldehyde and acetate production decreased by 94% and 61%, respectively, relative to the 15 × *cbbm* strain. These modifications did not affect glycerol production at 0.05 h^−1^ but caused a 4.6 fold higher glycerol production per amount of biomass in fast-growing (0.29 h^−1^) anaerobic batch cultures than observed for the 15 × *cbbm* strain. In another strategy, the promoter of *ANB1*, whose transcript level positively correlated with growth rate, was used to control PRK synthesis in a 2 × *cbbm* strain. At 0.05 h^−1^, this strategy reduced acetaldehyde and acetate production by 79% and 40%, respectively, relative to the 15 × *cbbm* strain, without affecting glycerol production. The maximum growth rate of the resulting strain equalled that of the reference strain, while its glycerol production was 72% lower.

**Conclusions:**

Acetaldehyde and acetate formation by slow-growing cultures of engineered *S. cerevisiae* strains carrying a PRK/RuBisCO bypass of yeast glycolysis was attributed to an in vivo overcapacity of PRK and RuBisCO. Reducing the capacity of PRK and/or RuBisCO was shown to mitigate this undesirable byproduct formation. Use of a growth rate-dependent promoter for PRK expression highlighted the potential of modulating gene expression in engineered strains to respond to growth-rate dynamics in industrial batch processes.

**Supplementary Information:**

The online version contains supplementary material available at 10.1186/s13068-023-02329-9.

## Background

With an estimated annual production of 103 billion litres in 2021 [1], bioethanol remains the largest-volume product of industrial biotechnology. Although use of alternative microbial production platforms based on autotrophic conversion of carbon monoxide and/or carbon dioxide is being intensively explored [2], ethanol is still predominantly produced via fermentation of plant-derived glucose and sucrose by the yeast *Saccharomyces cerevisiae* [3]. In these yeast-based processes, costs of the carbohydrate feedstock make up more than half of the total production costs [4]. Therefore, maximizing the ethanol yield on feedstock is an economically important goal.

Alcoholic fermentation of glucose and sucrose by *S. cerevisiae* starts with the ATP-yielding Embden–Meyerhof glycolytic pathway, whose product pyruvate is converted to ethanol and carbon dioxide by the combined action of pyruvate decarboxylase and alcohol dehydrogenase [5]. This native yeast pathway for sugar fermentation is redox-neutral and generates ATP. Its maximum product yield of 2 mol of ethanol per mole of glucose corresponds to the theoretical maximum yield based on conservation laws [6]. Indeed, near-theoretical ethanol yields on glucose were demonstrated during prolonged cultivation of *S. cerevisiae* at near-zero specific growth rates in anaerobic retentostat cultures [7]. In such non-growing cultures, sugar is almost exclusively used to generate ATP for cellular maintenance rather than for supporting yeast growth. Ethanol production in industrial fermentation processes is, however, accompanied by anaerobic growth, which requires the use of sugar for biomass formation. In addition, formation of *S. cerevisiae* biomass from sugars and ammonium or urea leads to a net reduction of NAD^+^ to NADH [8]. Under anaerobic conditions, re-oxidation of this ‘surplus’ NADH occurs by reduction of dihydroxyacetone-phosphate to glycerol-3-phosphate, which, upon hydrolysis, yields glycerol and phosphate [9]. In non-engineered *S. cerevisiae* strains, glycerol formation can account for a loss of up to 4% of the carbohydrate feedstock [10].

**Fig. 1:**
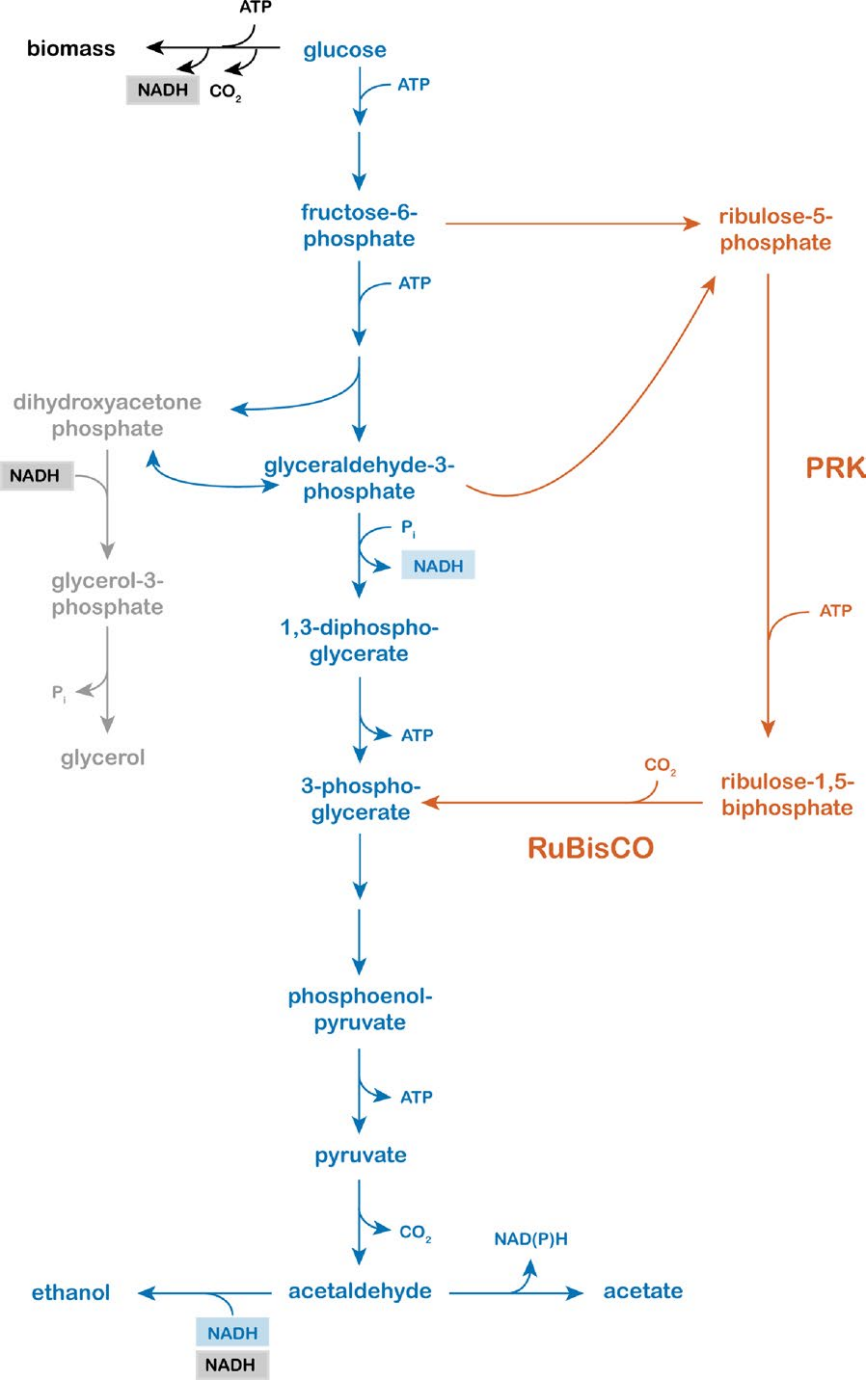
Simplified schematic representation of ethanol and biomass formation by an engineered strain of Saccharomyces cerevisiae by heterologous expression of genes encoding the Calvin-cycle enzymes PRK and RuBisCO. Black: biosynthetic reactions with a net input of ATP and a net production of CO_2_ and NADH. Blue: redox-neutral conversion of glucose to ethanol by glycolysis, pyruvate decarboxylase and alcohol dehydrogenase. Blue: NAD(P)H-dependent conversion of acetaldehyde into acetate. Orange: non-oxidative pentose phosphate pathway, heterologously expressed PRK/RuBisCO pathway and subsequent NADH-requiring ethanol formation. Grey: native glycerol reduction pathway

Multiple metabolic engineering strategies have been designed to reduce glycerol formation and, thereby, improve ethanol yield (see [11] for a recent review). The present study focuses on a strategy based on a bypass of the oxidative reaction in glycolysis that involves the Calvin-cycle enzymes phosphoribulokinase (PRK) and ribulose-1,5-bisphosphate carboxylase (RuBisCO, Fig. 1). In a first proof-of-concept for this strategy, Guadalupe-Medina et al. [12], who co-expressed a spinach PRK gene, a bacterial RuBisCO-encoding *cbbM* gene and the *E. coli* chaperonin-encoding *groEL* and *groES* genes in *S. cerevisiae*. In slow-growing sugar-limited chemostat cultures (0.05 h^−1^), the glycerol yield on sugar of an engineered strain carrying these modifications was 90% lower than that of the reference strain, while its ethanol yield on sugar was 10% higher.

Building on the initial proof-of-principle experiments, the metabolic network of PRK/RuBisCO-expressing *S. cerevisiae* strains was further engineered to improve specific growth rates in anaerobic batch cultures [13]. Overexpression of the structural genes for enzymes of the non-oxidative pentose-phosphate pathway (non-ox PPP↑; p*TDH3-RPE1*, p*PGK1-TKL1*, p*TEF1-TAL1*, p*PGI1-NQM1*, p*TPI1-RKI1* and p*PYK1-TKL2*) was implemented to increase supply of ribulose-5-phosphate, while multiple copies of a bacterial *cbbm* RuBisCO expression cassette were integrated in the yeast genome to improve conversion of ribulose-5-bisphosphate into 3-phosphoglycerate. Moreover, *GPD2*, which encodes an isoenzyme of NAD^+^-dependent glycerol-3-phosphate dehydrogenase, was deleted to reduce competition for biosynthetic NADH in fast-growing cultures. Finally, expression of the spinach PRK gene from the anaerobically inducible *DAN1* promoter [14, 15] limited toxic effects of PRK during aerobic pre-cultivation. The resulting PRK/RuBisCO-synthesizing *S. cerevisiae* strain (IMX1489; Δ*gpd2*, non-ox PPP↑, p*DAN1*-*prk*, 15 × *cbbm*, *GroES/GroEL*) showed essentially the same maximum growth rate on glucose as a non-engineered reference strain in anaerobic batch bioreactors while showing an 86% lower glycerol yield and an over 10% higher ethanol yield than the reference strain [13].

The results obtained with fast-growing anaerobic batch cultures of an optimized PRK/RuBisCO strain were promising. However, in industrial ethanol fermentation, the specific growth rate is not high throughout the process but, instead, decreases as the ethanol concentration reaches inhibitory levels [16] and/or non-sugar nutrients are depleted. The goal of the present study was therefore to investigate performance of a PRK/RuBisCO-expressing strain that was optimized for high ethanol yield and fast growth (specific growth rate of 0.29 h^−1^) at submaximal specific growth rates (0.05, 0.1 and 0.25 h^−1^) in anaerobic chemostat cultures. As these slow-growing cultures showed substantial production of acetaldehyde and acetate, we explored metabolic engineering strategies to reduce or prevent an apparent in vivo overcapacity of the key enzymes of the PRK/RuBisCO bypass and, thereby, of the formation of these undesirable byproducts.

## Results

### Acetaldehyde and acetate as key byproducts in slow-growing anaerobic cultures of a PRK/RuBisCO-synthesizing S. cerevisiae strain

To investigate the impact of the specific growth rate on product yields of a PRK/RuBisCO-synthesizing *S. cerevisiae* strain, anaerobic glucose-limited chemostat cultures of strain IMX1489 (Δ*gpd2*, non-ox PPP↑, p*DAN1*-*prk*, 15 × *cbbm*, *GroES/GroEL* [13]; non-ox PPP↑ indicates overexpression of native *S. cerevisiae* genes encoding enzymes of the non-oxidative pentose-phosphate pathway) were grown at dilution rates of 0.05 h^−1^, 0.1 h^−1^ and 0.25 h^−1^. Biomass and product yields of these cultures were compared with those of cultures of the congenic reference strain IME324, which carried none of the genetic modifications introduced into strain IMX1489. In addition, exponentially growing anaerobic batch cultures of the two strains were compared.

**Fig. 2:**
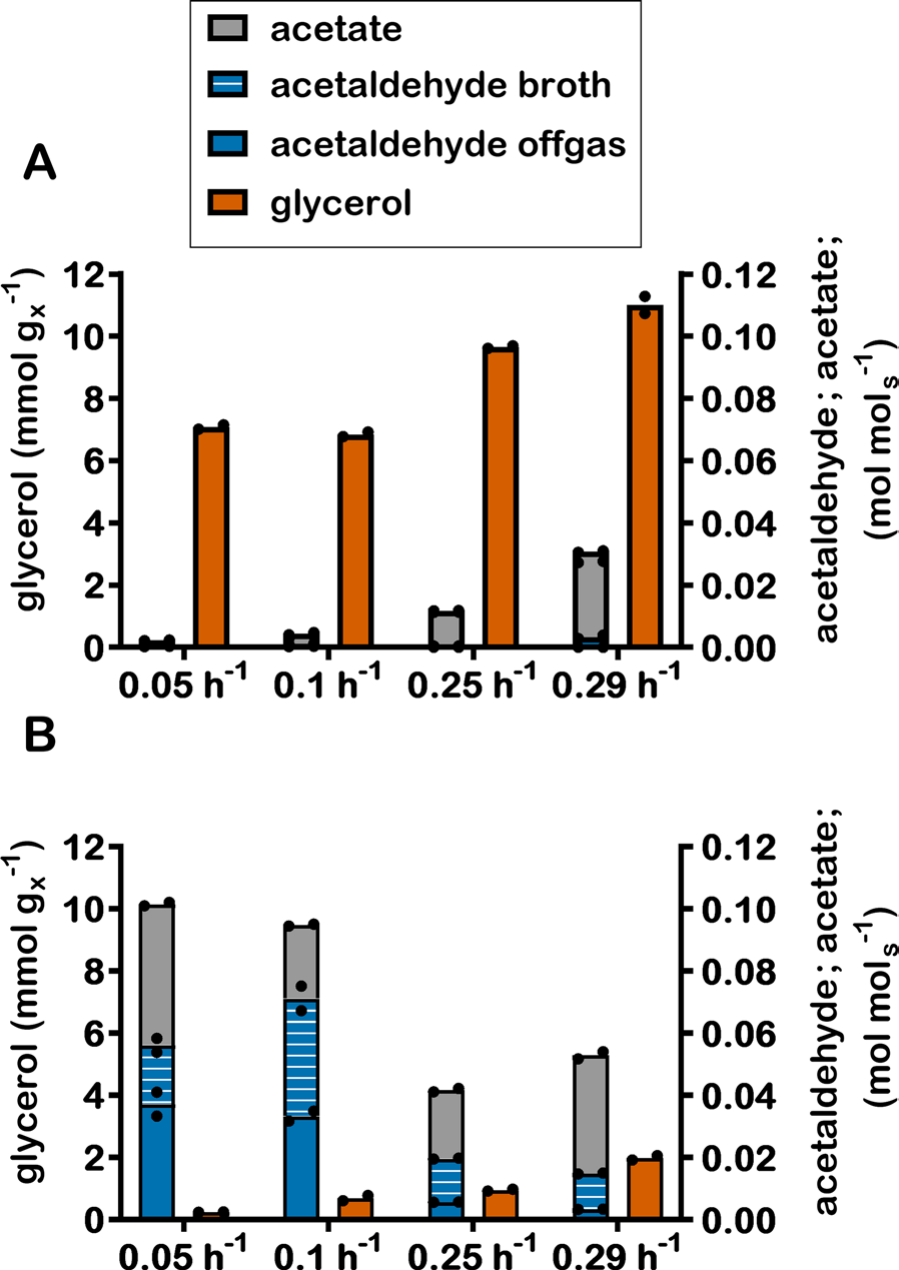
Yields of acetaldehyde and acetate on glucose and stoichiometric relationships between glycerol production and formation of biomass (denoted by x) in anaerobic chemostat cultures of *S. cerevisiae* strains IME324 (A: reference strain lacking PRK/RuBisCO bypass) and IMX1489 (B; Δ*gpd2*, non-ox PPP↑, p*DAN1*-*prk*, 15 × *cbbm*, *GroES/GroEL*) at 0.05 h^−1^, 0.1 h^−1^ and at 0.25 h^−1^ and anaerobic batch cultures at maximum specific growth rates (0.31 h^−1^ and 0.29 h^−1^). Values represent means and individual values of measurements on independent steady-state duplicate cultures

At all three dilution rates, chemostat cultures of the PRK/RuBisCO-synthesizing strain IMX1489 produced much less glycerol per amount of biomass formed than the reference strain (Fig. 2, Table 1). This observation showed that, at all three dilution rates, the PRK/RuBisCO-pathway replaced glycerol formation as main mechanism for oxidation of ‘surplus’ NADH derived from biosynthesis [8]. The relative impact of the PRK/RuBisCO pathway on glycerol formation was largest at low specific growth rates (Fig. 2, Table 1). At a dilution rate of 0.05 h^−1^, the amount of glycerol produced per amount of biomass by strain IMX1489 was only 3.5% of that of the reference strain, as compared to 10% at a dilution rate of 0.25 h^−1^ and 18% in exponentially growing batch cultures (Tables 1, 2).

**Table 1:** Yields of biomass, acetaldehyde, acetate and ethanol on glucose and ratio of glycerol production and biomass (x) formation in anaerobic chemostat cultures of *S. cerevisiae* strains IME324 (reference strain lacking PRK/RuBisCO bypass) and IMX1489 (Δ*gpd2*, non-ox PPP↑, p*DAN1*-*prk*, 15 × *cbbm*, *GroES/GroEL*) at 0.05 h^−1^, 0.1 h^−1^ and at 0.25 h^−1^

Strain	IME324			IMX1489		
Relevant genotype	*GPD2*			Δ*gpd2* p*DAN1*-*prk* 15 × *cbbm*		
Dilution rate (h^−1^)	0.05	0.1	0.25	0.05	0.1	0.25
Biomass/glucose (g_x_/g)	0.084 ± 0.002	0.096 ± 0.004	0.093 ± 0.001	0.078 ± 0.001	0.090 ± 0.001	0.093 ± 0.001
Ethanol/glucose (mol/mol)	1.539 ± 0.010	1.590 ± 0.039	1.487 ± 0.001	1.585 ± 0.010	1.574 ± 0.018	1.630 ± 0.007
Acetaldehyde/glucose (mol/mol)	0.001 ± 0.000	0.001 ± 0.000	0.000 ± 0.000	0.056 ± 0.002	0.071 ± 0.003	0.020 ± 0.000
Acetate/glucose (mol/mol)	0.002 ± 0.000	0.003 ± 0.001	0.011 ± 0.000	0.045 ± 0.001	0.024 ± 0.000	0.022 ± 0.001
Glycerol/glucose (mol/mol)	0.108 ± 0.001	0.118 ± 0.003	0.161 ± 0.000	0.004 ± 0.000	0.011 ± 0.002	0.016 ± 0.001
Glycerol/biomass (mmol/g_x_)	7.10 ± 0.10	6.85 ± 0.10	9.65 ± 0.06	0.25 ± 0.00	0.70 ± 0.12	0.96 ± 0.04
Electron recovery (%)	95–95	98–102	98–98	95–95	96–97	97–98

Values represent average ± mean deviation of measurements on independent steady-state duplicate cultures. Electron recoveries were calculated as balances of degree of reduction of substrates and products [21]

**Table 2:** Anaerobic bioreactor batch cultures of *S. cerevisiae* strains carrying different genetic modifications. Specific growth rates, electron recoveries and yields of ethanol, acetaldehyde and acetate on glucose and ratio between glycerol and biomass (x) formation were calculated from at least 6 sampling points in the exponential growth phase

Strain	IME324	IMX1489	IMX2736	IMX2701	IMX2593	IMX2608
Relevant genotype	*GPD2*	Δ*gpd2* p*DAN1*-*prk* 15 × *cbbm*	Δ*gpd2* p*DAN1*-*prk* 2 × *cbbm*	Δ*gpd2* p*DAN1*-*prk*-PEST 2 × *cbbm*	Δ*gpd2* p*DAN1*-*prk*-19aa 2 × *cbbm*	Δ*gpd2* p*ANB1*-*prk* 2 × *cbbm*
Biomass/glucose (g_x_/g)	0.091 ± 0.001	0.091 ± 0.001	0.095 ± 0.001	0.090 ± 0.001	0.094 ± 0.002	0.094 ± 0.001
µ_max_ (h^−1^)	0.32 ± 0.01	0.29 ± 0.00	0.29 ± 0.01	0.25 ± 0.00	0.29 ± 0.00	0.29 ± 0.00
Ethanol/glucose (mol/mol)	1.473 ± 0.046	1.601 ± 0.001	1.626 ± 0.008	1.544 ± 0.001	1.602 ± 0.007	1.647 ± 0.011
Acetaldehyde/glucose (mol/mol)	0.003 ± 0.001	0.015 ± 0.000	0.007 ± 0.001	0.001 ± 0.000	0.003 ± 0.002	0.004 ± 0.001
Acetate/glucose (mol/mol)	0.027 ± 0.000	0.038 ± 0.001	0.034 ± 0.001	0.013 ± 0.001	0.019 ± 0.002	0.025 ± 0.002
Glycerol/glucose (mol/mol)	0.202 ± 0.004	0.033 ± 0.001	0.037 ± 0.002	0.153 ± 0.003	0.086 ± 0.002	0.052 ± 0.005
Glycerol/biomass (mmol/g_x_)	11.3 ± 0.55	1.99 ± 0.07	1.86 ± 0.07	9.08 ± 0.06	5.05 ± 0.20	3.12 ± 0.25
Electron recoveries (%)	100–101	98–98	100–101	101–102	100–101	100–100

Biomass yield was determined based on the measured concentrations of biomass and glucose of the first and last timepoint. Values represent averages ± mean deviations of measurements on independent duplicate cultures for each strain. Electron recoveries were calculated as balances of degree of reduction of substrates and products [21]

Despite the near-complete elimination of glycerol formation in chemostat cultures of strain IMX1489 grown at 0.05 h^−1^, the ethanol yield on glucose in these cultures was only 3.0% higher than observed in similar cultures of the reference strain. This difference was smaller than observed in chemostat cultures grown at a dilution rate of 0.25 h^−1^ and in exponentially growing batch cultures (9.5% and 8.7%, respectively, Tables 1, 2). At all three dilution rates, strain IMX1489 showed a higher acetate yield on glucose than the reference strain IME324, with the highest relative difference being observed at 0.05 h^−1^. At this low dilution rate, acetate was a minor byproduct in cultures of the reference strain (0.002 mol acetate per mol glucose), while strain IMX1489 showed a 22-fold higher acetate yield (0.045 mol acetate per mol glucose) (Table 1, Fig. 2). The smallest difference was found in exponentially growing batch cultures, in which strain IMX1489 showed a on only 40% higher acetate yield than the reference strain (Table 2).

A distinct smell of the off-gas of chemostat cultures indicated that strain IMX1489 produced acetaldehyde. To quantify this volatile metabolite (boiling temperature 20 °C, [17]), culture broth was rapidly sampled into a 2,4-dinitrophenylhydrazine (2,4-DNPH) solution [18] and acetaldehyde in the gas phase was measured after sparging bioreactor off-gas through a 2,4-DNPH solution. Low acetaldehyde yields on glucose (≤ 0.001 mol acetaldehyde per mol glucose; Table 1) were found in chemostat cultures of the reference strain IME324. Acetaldehyde yields of strain IMX1489 were much higher, with the highest values observed at dilution rates of 0.05 h^−1^ and 0.10 h^−1^ (0.056 and 0.071 mol acetaldehyde (mol glucose)^−1^, respectively). In these cultures, concentrations of acetaldehyde of up to 6.5 mM were measured in the culture broth (Additional file 1). This concentration is within the toxicity range for yeasts [19] and may explain why biomass yields of strain IMX1489 at these dilution rates were 6 to 7% lower than those of the reference strain IME324. Acetaldehyde yields on glucose of chemostat cultures at 0.25 h^−1^ and anaerobic batch cultures of strain IMX1489 were lower (0.020 mol (mol glucose)^−1^ and 0.015 mol (mol glucose)^−1^, respectively) than observed in slow-growing chemostat cultures. Biomass yields in those cultures were similar to those of the control strain. To investigate whether acetate and acetaldehyde production by the PRK/RuBisCO-synthesizing strain depended on the nutrient-limitation regime, anaerobic nitrogen-limited chemostat cultures were grown at 0.1 h^−1^. Also in those cultures, strain IMX1489 showed a much higher acetaldehyde yield on glucose than the reference strain IME324 (Additional file 6: Figure S1).

Based on the high yields of acetate and acetaldehyde in anaerobic cultures of the PRK/RuBisCO-synthesizing strain IMX1489, we hypothesized that the rate of pyruvate generation via the PRK/RuBisCO bypass (Fig. 1) exceeded the rate of ‘surplus’ NADH formation in biosynthesis. In such a scenario, limited availability of NADH would prevent complete reduction of acetaldehyde generated in the irreversible pyruvate-decarboxylase reaction by NADH-dependent alcohol dehydrogenase. The excess acetaldehyde could then either be excreted or converted to acetate by (NAD(P)^+^-dependent acetaldehyde dehydrogenases (Ald6, Ald5 and Ald4 [20]). This hypothesis is consistent with the observed high acetaldehyde and acetate yields in slow-growing cultures since, under the assumption of a constant biomass composition, the biomass-specific rate of NADH formation in biosynthesis is proportional to the specific growth rate [8].

### Reduced acetaldehyde and acetate production in strains with a lower copy number of the RuBisCO-encoding expression cassette

**Fig. 3:**
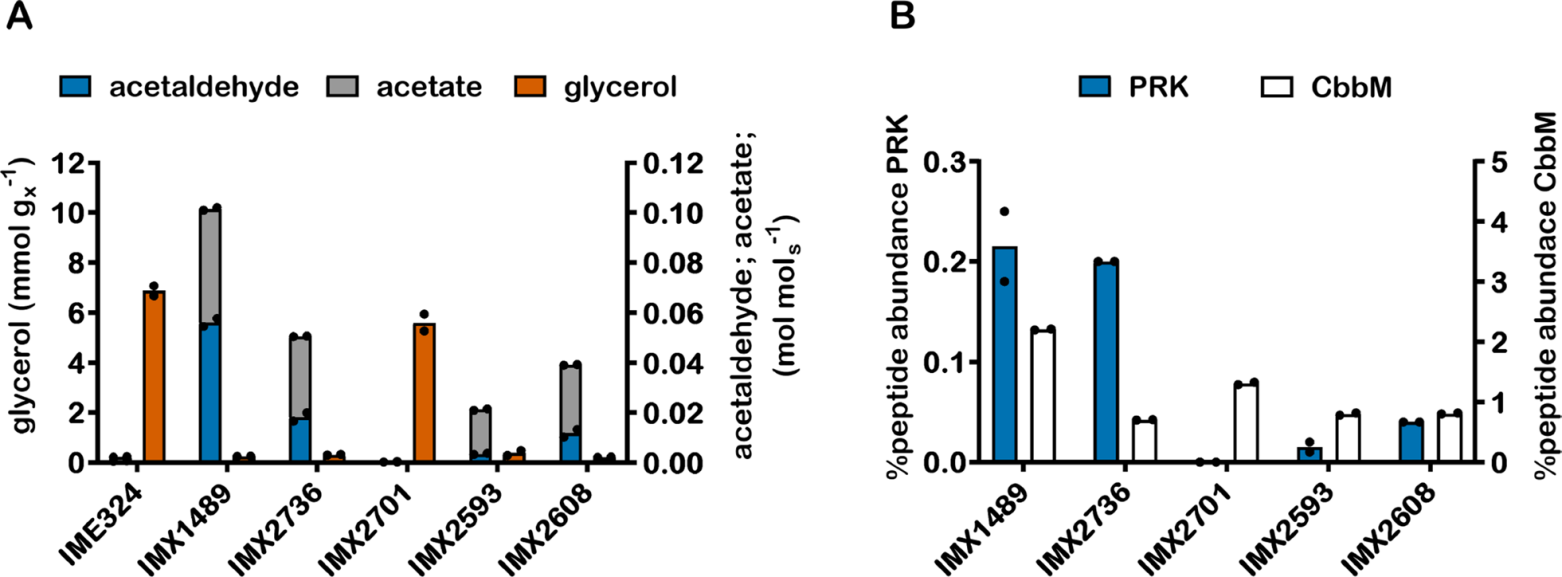
Glycerol, acetate and acetaldehyde production (**A**) and PRK and CbbM-derived peptide abundance determined by LC–MS and displayed as the percentage of PRK or CbbM protein in the total protein pool (**B**) in anaerobic chemostat cultures of IME324 (reference strain lacking PRK/RuBisCO bypass), IMX1489 (Δ*gpd2*, non-ox PPP↑, p*DAN1*-*prk*, 15 × *cbbm*, *GroES/GroEL*), IMX2736 (Δ*gpd2*, non-ox PPP↑, p*DAN1*-*prk*, 2 × *cbbm*, *GroES/GroEL*), IMX2701 (Δ*gpd2*, non-ox PPP↑, p*DAN1*-*prk*-*CLN2*_PEST_, 2 × *cbbm*, *GroES/GroEL*), IMX2593 (Δ*gpd2*, non-ox PPP↑, p*DAN1*-*prk*-19aa, 2 × *cbbm*, *GroES/GroEL*) and IMX2608 (Δ*gpd2*, non-ox PPP↑, p*ANB1*-*prk*, 2 × *cbbm*, *GroES/GroEL*) at a dilution rate of 0.05 h^−1^. Values represent means and individual values of measurements on independent steady-state duplicate cultures. Electron recoveries were calculated as balances of degree of reduction of substrates and products [21]

To maximize the positive impact of the engineered PRK/RuBisCO-bypass on ethanol yield, production of acetate and acetaldehyde, resulting from a too high activity of this pathway, should be minimized. In strain IMX1489, multiple copies [22] of the *cbbm* expression cassette were introduced in tandem to facilitate amplification of the number of *cbbm* expression cassettes by homologous recombination. Such homologous recombination can also lead to a reduction of the *cbbm* copy number but, since adjacent copies of the *cbbm* cassette were divergently oriented, could not lead to a copy number below two. Short-read whole-genome sequencing (WGS) and long-read sequencing estimated the copy number in strain IMX1489 at 13 and 15 copies, respectively [23]. However, strain IMX2736 and other strains derived from IMX2288, which was obtained by removing the gRNA plasmid from strain IMX1489, contained only 2 copies. Strain IMX2736, which did not reveal mutations in open reading frames relative to strain IMX1489, was therefore used to study the impact of a reduced *cbbm* copy number on byproduct formation. In slow-cultures, the sevenfold lower copy number of *cbbm* in strain IMX2736 coincided with a threefold lower abundance of the CbbM protein than in strain IMX1489 (0.70 ± 0.01% and 2.20 ± 0.01% of total protein, respectively, in duplicate anaerobic chemostats of each strain grown at 0.05 h^−1^). In contrast, PRK abundance was not significantly different (Fig. 3B). In these slow-growing chemostat cultures, acetaldehyde and acetate yields of strain IMX2736 were 67% and 29% lower, respectively, than those of strain IMX1489 (p*DAN1*-*prk* 15 × *cbbm*), while ethanol yield was 4% higher (Table 3). The 12.8% higher biomass yield of strain IMX2736 may reflect alleviation of acetaldehyde toxicity.

**Table 3:** Yields of biomass, acetaldehyde, acetate and ethanol on glucose and ratio between glycerol production and biomass formation in anaerobic glucose-limited chemostat cultures of *S. cerevisiae* strains carrying different genetic modifications at a dilution rate of 0.05 h^−1^

Strain	IME324	IMX1489	IMX2736	IMX2701	IMX2593	IMX2608
Relevant genotype	*GPD2*	Δ*gpd2* p*DAN1*-*prk* 15 × *cbbm*	Δ*gpd2* p*DAN1*-*prk* 2 × *cbbm*	Δ*gpd2* p*DAN1*-*prk*-PEST 2 × *cbbm*	Δ*gpd2* p*DAN1*-*prk*-19aa 2 × *cbbm*	Δ*gpd2* p*ANB1*-*prk* 2 × *cbbm*
Biomass/glucose (g_x_/g)	0.084 ± 0.002	0.078 ± 0.001	0.088 ± 0.002	0.085 ± 0.000	0.089 ± 0.002	0.090 ± 0.000
Ethanol/glucose (mol/mol)	1.539 ± 0.010	1.585 ± 0.010	1.648 ± 0.005	1.549 ± 0.004	1.651 ± 0.034	1.626 ± 0.002
Acetaldehyde/glucose (mol/mol)	0.001 ± 0.000	0.056 ± 0.002	0.018 ± 0.002	0.000 ± 0.000	0.004 ± 0.000	0.012 ± 0.002
Acetate/glucose (mol/mol)	0.002 ± 0.000	0.045 ± 0.001	0.032 ± 0.002	0.000 ± 0.000	0.018 ± 0.000	0.027 ± 0.000
Glycerol/glucose (mol/mol)	0.108 ± 0.001	0.004 ± 0.000	0.005 ± 0.000	0.086 ± 0.006	0.006 ± 0.002	0.003 ± 0.000
Glycerol/biomass (mmol/g_x_)	7.10 ± 0.10	0.25 ± 0.00	0.31 ± 0.01	5.60 ± 0.41	0.39 ± 0.14	0.21 ± 0.01
Electron recovery (%)	95–95	95–95	97–98	95–95	95–97	96–96

Values represent averages ± mean deviations of measurements on independent steady-state duplicate cultures. Electron recoveries were calculated as balances of degree of reduction of substrates and products [21]

**Fig. 4:**
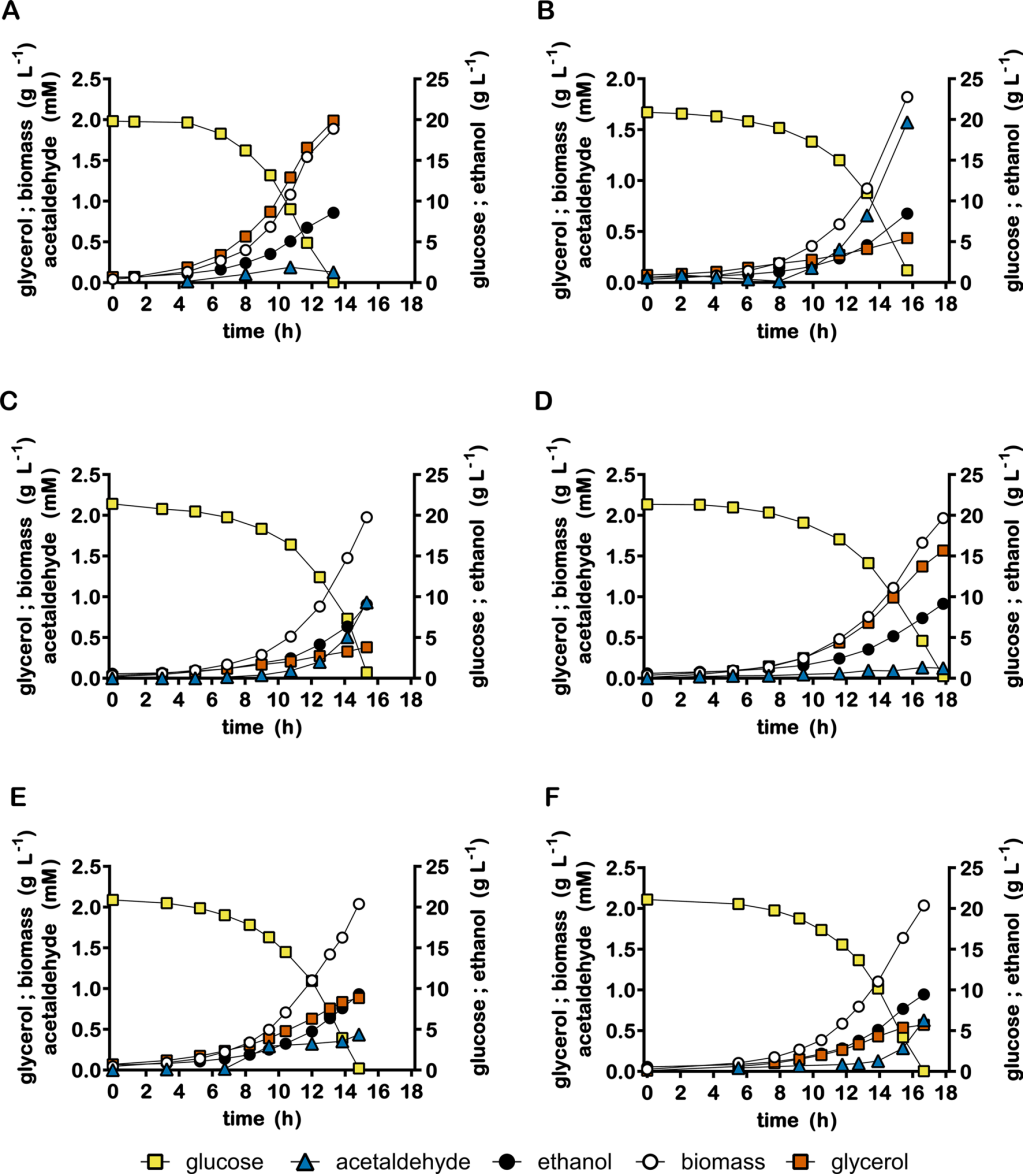
Growth, glucose consumption, ethanol formation, acetaldehyde formation and glycerol formation in anaerobic bioreactor batch cultures of *S. cerevisiae* strains IME324 (reference strain lacking PRK/RuBisCO bypass) (**A**), IMX1489 (Δ*gpd2*, non-ox PPP↑, p*DAN1*-*prk*, 15 × *cbbm, GroES/GroEL*)(**B**), IMX2736 (Δ*gpd2*, non-ox PPP↑, p*DAN1*-*prk*, 2 × *cbbm, GroES/GroEL*)(**C**), IMX2701 (Δ*gpd2*, non-ox PPP↑, p*DAN1*-*prk*-*CLN2*_PEST_, 2 × *cbbm, GroES/GroEL*)(**D**), IMX2593 (Δ*gpd2*, non-ox PPP↑, p*DAN1*-*prk*-19aa, 2 × *cbbm, GroES/GroEL*)(**E**) and IMX2608 (Δ*gpd2*, non-ox PPP↑, p*ANB1*-*prk*, 2 × *cbbm, GroES/GroEL*) (**F**) Cultures were grown anaerobically at pH 5 and at 30 °C on synthetic medium containing 20 g L^−1^ glucose. Representative cultures of independent duplicate experiments are shown, corresponding replicate of each culture shown in Additional file 7: Figure S3

Despite its sevenfold lower *cbbm* copy number, IMX2736 did not show a lower specific growth rate or higher glycerol yield per amount of biomass than strain IMX1489 in anaerobic batch cultures (Table 2, Fig. 4). In addition, acetate and acetaldehyde yields on glucose in anaerobic batch cultures of strain IMX2736 were 10.5% and 53% lower, respectively, than in similar cultures of strain IMX1489, while the ethanol yield was 1.5% higher (Table 2). As observed in slow-growing chemostat cultures, a lower production of acetaldehyde and acetate in strain IMX2736 coincided with a higher biomass yield on glucose relative to strain IMX1489.

### C-terminal tags for reducing PRK levels

C-terminal PEST sequences, which are rich in proline (P), glutamic acid (E), serine (S) and threonine (T), have been described to enhance proteasome-mediated protein turnover [24]. This regulation mechanism could, in principle, not only reduce PRK levels in growing cultures but, in particular, support fast PRK degradation once growth and protein synthesis in industrial processes slows down. Regulation of PRK activity at a post-translational level is also attractive because it is not expected to interfere with oxygen-dependent expression of *prk* from the p*DAN1* promoter, which was used to prevent toxicity of high levels of PRK during aerobic pre-cultivation [25].

When the DNA sequence encoding the 178-amino acid C-terminal *CLN2*_*PEST*_ sequence [24] was C-terminally fused to *prk* in strain IMX2701 (p*DAN1*-*prk-CLN2*_*PEST*_ 2 × *cbbm*), PRK levels were below the detection limit of the proteomics analysis (Fig. 3B). This extreme reduction of PRK protein levels relative to those in strain IMX2736 (p*DAN1*-*prk* 2 × *cbbm*) was reflected in a 19-fold and fivefold higher glycerol formation per amount of biomass in chemostat cultures grown at 0.05 h^−1^ and in anaerobic batch cultures, respectively (Tables 2, 3). Strain IMX2701 showed a 14% lower specific growth rate in anaerobic batch cultures than strain IMX2736 (Table 2, Fig. 4D). This observation is consistent with a limited capacity for anaerobic reoxidation of cytosolic NADH which, at the extremely low PRK level in strain IMX2701 (Fig. 3B), predominantly depends on sole remaining glycerol-3-phosphate isoenzyme Gpd1 in the engineered strains.

While constructing fusions of *prk* with the *CLN2*_*PEST*_ tag, a mistake during strain construction yielded a fusion of *prk* with a frameshifted PEST-sequence. This mistake was only discovered after the resulting strain IMX2593 had been tested in bioreactor cultures and led to a 19-amino acid extension of PRK. This extension, which we labelled -19aa, showed no notable sequence similarity with the *CLN2*_*PEST*_ sequence (Additional file 6: Table S2). However, chemostat cultures of strain IMX2593 (p*DAN1*-*prk*-19aa 2 × *cbbm*) grown at 0.05 h^−1^ showed a 13-fold lower PRK protein level than strain IMX2736 (p*DAN1*-*prk* 2 × *cbbm*). Despite this strongly reduced PRK protein level, strain IMX2593 still showed an 18-fold lower glycerol production per amount of biomass than the reference strain IME324. Moreover, acetaldehyde and acetate yields on glucose in the chemostat cultures of strain IMX2593 were 81% and 45% lower, respectively, than in corresponding cultures of strain IMX2736. In exponentially growing batch cultures of strain IMX2593, glycerol formation per amount of biomass was 30% higher than in batch cultures of IMX2736, but still 55% lower than in batch cultures of the reference strain (IME324) (Table 2, Fig. 4E).

To explore how the 19-aa tag affected protein levels, it was fused to the C-terminus of eGFP. The resulting yeast strain showed an almost twofold lower fluorescence signal (Additional file 7: Figure S2; 0 min) than a strain expressing non-tagged eGFP gene. Both tagged and non-tagged eGFP remained stable upon addition of the protein-synthesis inhibitor cycloheximide [26] (Additional file 7: Figure S2). In contrast, a strain expressing an eGFP-*CLN2*_*PEST*_ fusion, whose steady-state fluorescence was sevenfold lower than that of a strain expressing non-tagged GFP, showed a fast decline of fluorescence after cycloheximide addition (Additional file 7: Figure S2). These results indicated that the 19aa tag did not substantially affect protein degradation kinetics.

### Promoter selection for PRK results in reduced overflow metabolism

While the serendipitously obtained 19aa C-terminal tag decreased PRK protein levels and formation of acetaldehyde and acetate, this mode of regulation of PRK synthesis is essentially static. Ideally, in vivo activity of the PRK/RuBisCO-pathway should be dynamically adapted to the rate of growth-coupled NADH formation in biosynthesis. To enable adaptation of *prk* expression to specific growth rate while retaining regulation of *prk* expression by oxygen [14, 15], we searched for an *S. cerevisiae* promoter that would support induction under anaerobic conditions as well as growth rate-dependent transcription.

**Fig. 5:**
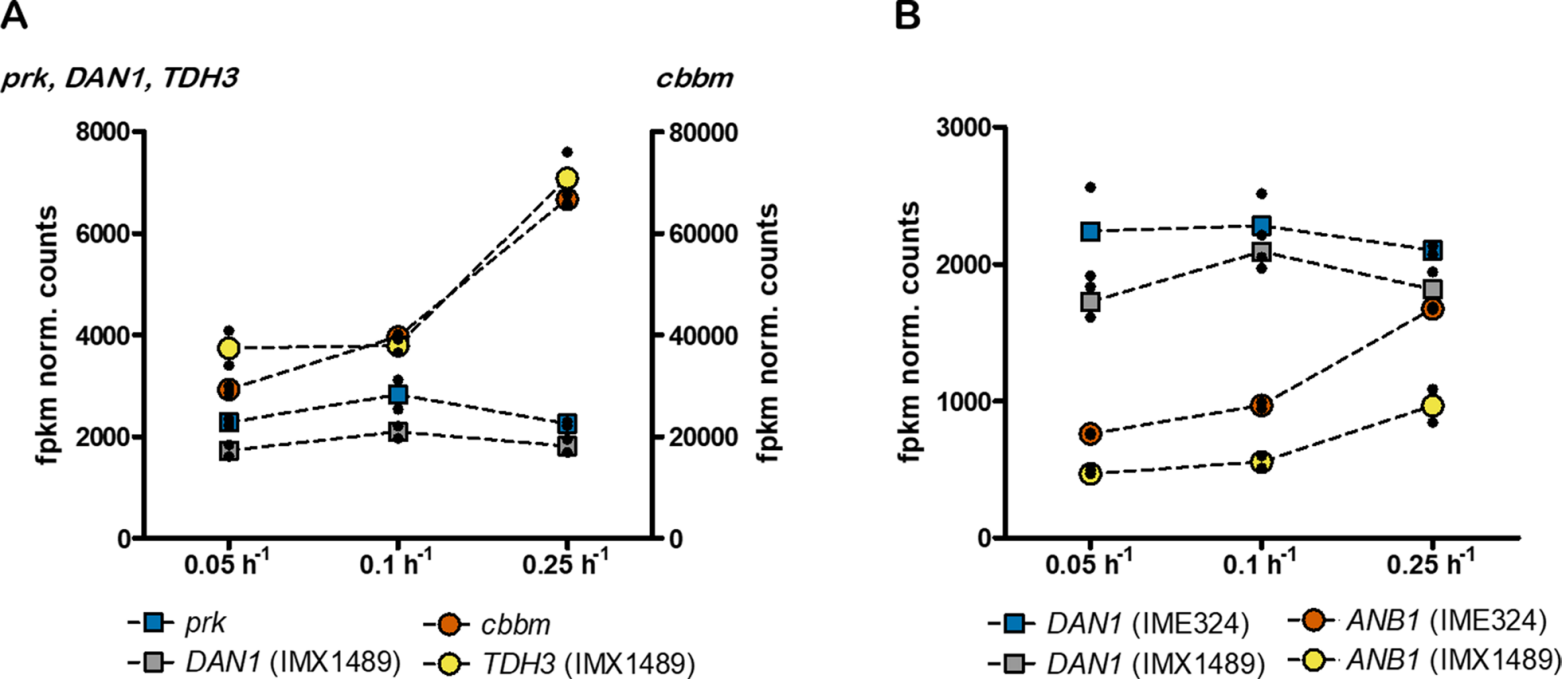
fpkm normalized counts of *prk*, *DAN1*, *cbbm* and *TDH3* obtained from transcriptome data of chemostat cultures of strain IMX1489 (Δ*gpd2*, non-ox PPP↑, p*DAN1*-*prk*, 15 × cbbm, *GroES/GroEL*) (**A**) and fpkm normalized counts of *DAN1* and *ANB1* obtained from transcriptome data of chemostat cultures of strain IMX1489 and IME324 (reference strain lacking PRK/RuBisCO bypass) (**B**) at dilution rates of 0.05 h^−1^, 0.1 h^−1^ and 0.25 h^−1^. Values represent means and individual values of measurements on independent steady-state duplicate cultures

In anaerobic, glucose-limited chemostat cultures of strains IMX1489 and IME324 grown at 0.05, 0.1 and 0.25 h^−1^, transcript levels of *DAN1* and *prk* expressed from the *DAN1* promoter did not show a significant correlation with specific growth rate (Fig. 5). Transcriptome data from these cultures were therefore analysed to identify genes whose anaerobic transcript levels positively correlated with specific growth rate and whose transcript level at 0.25 h^−1^ was at least 50% of the transcript level of *prk* observed upon its expression from the *DAN1* promoter. The search was further narrowed by excluding genes that were also expressed in aerobic cultures of the congenic reference strain CEN.PK113-7D (data set of Regenberg et al. [27]; Fig. 5). This search yielded only *ANB1*, which encodes a translation initiation factor [28]. Expression of *prk* from a 1000 bp *ANB1* promoter fragment yielded strain IMX2608 (*Δgpd2*, non-ox PPP↑, p*ANB1*-*prk*, 2 × *cbbm*, *GroES*/*GroEL*). In anaerobic chemostat cultures grown at 0.05 h^−1^, acetaldehyde and acetate yields on glucose of this strain were 36% and 15% lower than in corresponding cultures of strain IMX2736.

In anaerobic bioreactor batch cultures, strain IMX2608 showed a twofold lower PRK protein level than strain IMX2736, which expressed *prk-19aa* (Additional File 4). In these batch cultures, the specific growth rates of the two strains were not significantly different. Acetaldehyde and acetate yields of strain IMX2608 in anaerobic batch cultures were approximately 40% and 25% lower than in those of strain IMX2736 and not significantly different from those in batch cultures of the reference strain IME324 (Table 2). While the glycerol yield per amount of biomass of strain IMX2608 was 57% higher than in cultures of strain IMX 2736, it was still 72% lower than observed in batch cultures of the reference strain IME324 (Table 2, Fig. 4F).

## Discussion

An engineered non-oxidative glycolytic bypass mediated by the Calvin-cycle enzymes PRK and RuBisCO can replace glycerol formation as main mechanism for re-oxidation of biosynthesis-derived NADH in anaerobic *S. cerevisiae* cultures and, thereby, improve ethanol yields on sugar [13]. Our results show that, especially at low specific growth rates, strains carrying the PRK/RuBisCO glycolytic bypass produced more acetaldehyde and acetate than a reference strain. This enhanced byproduct formation was hypothesized to reflect an imbalance between in vivo activity of the PRK/RuBisCO bypass and generation of NADH in biosynthesis. Consistent with this hypothesis, reduction of PRK and/or RuBisCO synthesis by engineered strains led to a lower production of acetaldehyde and acetate. Slow-growing anaerobic cultures of a similarly engineered *S. cerevisiae* strain were recently shown to co-ferment glucose and sorbitol [23]. Since sorbitol co-fermentation required a higher flux through the PRK/RuBisCO bypass, these results are also in line with an ‘overcapacity’ of this pathway during slow anaerobic growth on glucose.

As generation of acetaldehyde and acetate goes at the expense of maximum achievable ethanol yield gains of the PRK/RuBisCO strain, the observed byproduct formation is undesirable for industrial application. Furthermore, slow-growing cultures of a PRK/RuBisCO strain that carried 15 copies of the expression cassette for RuBisCO reached acetaldehyde concentrations that were within the toxicity range for yeast [19]. Loss of carbon to acetaldehyde and acetate goes at the expense of the ethanol yield on sugar and, in addition, production of acetaldehyde in large-scale processes may raise environmental [29] and health [30] issues. We therefore explored metabolic engineering strategies to mitigate byproduct formation.

Reducing the copy number of the expression cassette for RuBisCO led to lower acetaldehyde and acetate production in slow-growing anaerobic cultures and, concomitantly, a higher ethanol yield on glucose (strain IMX2736; Fig. 3, Table 3). Further reduction of acetaldehyde and acetate production was tested by reducing the PRK protein level. One of the strategies used for decreasing PRK levels was based on the serendipitously discovered 19aa tag, which was found to reduce acetaldehyde and acetate production both in fast and slow-growing cultures (strain IMX2593; Fig. 3, Tables 2, 3). However, this engineering strategy, resulting in ‘static’ regulation of *prk* expression, also led to higher glycerol yields in fast-growing cultures (Table 2, Fig. 4), thus reflecting a trade-off between performance at low and high specific growth rates.

The 19-aa tag reduced PRK protein level without a need to change the promoter from which *prk* gene was expressed. Conserving transcriptional regulation of *prk* by an oxygen-responsive promoter was important, as it prevents toxicity of PRK in aerobic pre-cultures [13, 25]. Of the 19 nucleotide triplets that encode the 19aa tag, four occur at a frequency that is below 50% of that of the most frequently occurring synonymous triplet (Additional file 6: Table S3). However, it cannot be excluded that factors other than codon usage cause the reduced protein abundance of PRK and GFP observed upon addition of the 19aa tag. Resolving this issue would require analyses of protein and transcript levels, as well as protein activity in strains carrying C-terminal tags with different codon usage. Irrespective of the responsible mechanism, tuning of protein levels and/or activity by C-terminal tagging may be a simple, flexible strategy in scenarios that require conservation of transcriptional regulation of an expression cassette, for example because few suitable promoters are available. Flexibility of this approach could be increased by high-throughput screening of libraries of short C-terminal tags for different impacts on protein levels. In *E. coli*, different protein degradation tags were obtained by mutagenizing a known C-terminal tag based on the *Mesoplasma florum* tmRNA system [31]. In *S. cerevisiae*, the PEST-tag was used to similarly decrease protein stability [32–34] but, in the present study, a PRK-PEST fusion led to too low protein levels of PRK (Fig. 3).

Conditions in large-scale ethanol fermentation processes are inherently dynamic due to changes in the concentrations of sugar, nitrogen source, ethanol and other compounds [35]. In industrial contexts, the capacity of the PRK/RuBisCO bypass should therefore ideally be dynamically adapted to the actual rate of NADH generation in biosynthesis. The potential of such dynamic regulation strategies was demonstrated by expressing the *prk* gene from the promoter of *ANB1*, whose transcript levels in anaerobic chemostat cultures positively correlated with specific growth rate (Fig. 5; [28]). While yields of acetate and acetaldehyde in slow-growing cultures of the resulting *pANB1-prk* strain IMX2608 were not as low as in strain IMX2593, which is based on the ‘static’ regulation of PRK (Table 3), they were similar to those in fast-growing cultures of the non-engineered reference strain IME324 (Table 2). In addition, the p*ANB1-prk* strain showed a strong reduction of glycerol production and high ethanol yield in anaerobic batch cultures relative to this reference strain (Table 2).

Instead of relying on yeast promoters such as *ANB1*, *prk* expression might be coupled to synthetic regulatory circuits [36] that employ ligand-binding transcription factors as biosensors. Development of biosensor–promoter combinations with the required ligand sensitivity and dynamic range requires extensive optimization [37, 38], but offers unique possibilities to dynamically regulate abundance of key proteins. For example, biosensor-controlled expression of dCas9 [39], an mRNA-interference module [40] or activate auxin-mediated degradation [41], enables regulation at the level of transcription, translation or protein-turnover, respectively. Dynamic regulation of *prk* expression might thus, for example, be coupled to NADH availability via the NADH-responsive bacterial transcriptional repressor Rex [42–44] or to intracellular acetaldehyde concentration via the aldehyde-responsive *E. coli* transcriptional activator YqhC [45–47]. While these systems have been applied in bacterial hosts, they have not yet been implemented in anaerobic yeast cultures.

Byproduct formation in yeast strains engineered for reduced glycerol formation is not necessarily a unique characteristic of *S. cerevisiae* strains carrying a PRK/RuBisCO bypass. In an alternative strategy based on heterologous phosphoketolase and phosphotransacetylase [48, 49], acetyl-CoA is generated via acetyl-phosphate. The subsequent reduction of acetyl-CoA to acetaldehyde via acetylating acetaldehyde dehydrogenase requires NADH, while an additional NADH is required to further reduce acetaldehyde to ethanol. By analogy to the situation in PRK/RuBisCO-expression strains, a too high activity of phosphoketolase, phosphotransacetylase and/or acetylating acetaldehyde dehydrogenase could theoretically also lead to accumulation of acetaldehyde (and acetate). Yet another strategy for glycerol reduction relies on the heterologous pyruvate-formate lyase and acetylating acetaldehyde dehydrogenase [50, 51], which together convert pyruvate in into acetaldehyde and formate. While formation of acetaldehyde from glucose via this pathway is redox-cofactor neutral, its subsequent reduction to ethanol requires NADH. Slow anaerobic growth of engineered yeast strains that rely on this pathway for reoxidation of ‘surplus’ NADH from biosynthesis, combined with a high activity of pyruvate-formate lyase and acetylating acetaldehyde dehydrogenase, might therefore also result in acetaldehyde accumulation.

Redox-cofactor engineering strategies are typically designed based on pathway stoichiometry, which for organisms such as *S. cerevisiae* can be easily modelled by metabolic network models [52]. Our results underline the importance of also considering pathway kinetics and process dynamics in redox-cofactor based engineering studies and show the potential of dynamic regulation mechanisms to optimize microbial performance in industrial processes.

## Materials and methods

### Strains, media and maintenance

*S. cerevisiae* strains used in this study were derived from the CEN.PK lineage [53, 54]. Synthetic medium (SM) containing 3.0 g L^−1^ KH_2_PO_4_, 0.5 g L^−1^ MgSO_4_·7H_2_O, 5.0 g L^−1^ (NH_4_)_2_SO_4_, trace elements and vitamins was prepared as described previously [55]. Shake-flask, bioreactor-batch and glucose-limited chemostat cultures were grown on SM supplemented with 20 g L^−1^ glucose (SMD). In synthetic medium for nitrogen-limited chemostat cultivation [56], the concentration of (NH_4_)_2_SO_4_ was reduced to 1.0 g L^−1^, 5.3 g L^−1^ K_2_SO_4_ was added and the glucose concentration was increased to 59 g L^−1^. Anaerobic growth media were supplemented with ergosterol (10 mg L^−1^) and Tween 80 (420 mg L^−1^) [9]. Complex medium (YPD) contained 10 g L^−1^ Bacto yeast extract (Thermo Fisher Scientific, Waltham MA), 20 g L^−1^ Bacto peptone (Thermo Fisher Scientific) and 20 g L^−1^ glucose. *Escherichia coli* XL1-Blue cultures were grown on lysogeny broth (LB) [57] containing 10 g L tryptone (Brunschwig Chemie B.V., Amsterdam, The Netherlands), 5.0 g L^−1^ yeast extract and 10 g L^−1^ NaCl, where indicated supplemented with 100 mg L^−1^ ampicillin (Merck, Darmstadt, Germany). *E. coli* strains were grown overnight at 37 °C in 15-mL tubes containing 5 mL LB, shaken at 200 rpm in an Innova 4000 Incubator (Eppendorf AG, Hamburg, Germany). Agar (20 g L^−1^; Becton Dickinson, Breda, The Netherlands) was added to obtain solid-medium plates. *S. cerevisiae* plate cultures were incubated at 30 °C until colonies appeared (2–3 d), while *E. coli* plates were incubated overnight at 37 °C. Frozen stock cultures were prepared by freezing samples from fully grown batch cultures at -80 °C after addition of 30% (v/v) glycerol.

### Plasmid and cassette construction

Plasmids and oligonucleotide primers used in this study, the latter either HPLC or PAGE purified or desalted (DST), are listed in Table 4 and Additional file 6: Table S1, respectively. Phusion High-Fidelity DNA polymerase and Dreamtaq polymerase (Thermo Fisher Scientific) were used for PCR amplification of DNA fragments for further assembly and for diagnostic PCR reactions, respectively, according to the supplier’s instructions. PCR-amplified DNA fragments were purified from gels using the ZymoClean Gel DNA recovery kit (Zymoclean, D2004, Zymo Research, Irvine, CA) or directly from PCR reaction mixtures using a GeneJET PCR purification kit (Thermo Fisher Scientific) following supplier’s protocols. Plasmids were assembled with the in vitro Gibson method, using the HiFi DNA Assembly Master Mix (New England Biolabs, Ipswich, MA), essentially as recommended by the manufacturer but with a downscaled 5-µL reaction volume. XL-1 Blue cells were transformed by heat shock [58] with 1 µL of assembly mix. Assembled plasmids were isolated from *E. coli* cultures with a GenElute Plasmid kit (Sigma-Aldrich, St. Louis, MO) and used to transform *S. cerevisiae* strains. Yeast genomic DNA was isolated as described by Lõoke et al. [59].

**Table 4:** Plasmids used in this study

Plasmid	Characteristics	Origin
p426-*GPD*	2 µm ori, *URA3*, p*TDH3* -t*CYC1* (empty vector)	[63]
pROS10	2 µm ori*, KIURA3*, gRNA.*CAN1*-gRNA.*ADE2*	[61]
pUDR103	2 µm ori, *KIURA3*, p*SNR52*-gRNA.*SGA1*-t*SUP4*	[13]
pUDR760	2 µm ori, *KIURA3*, p*SNR52*-gRNA.*prk*-t*SUP4*	This study
pUDE046	2 µm ori, *TRP1*, p*GAL1*-*prk*-t*CYC1*	[12]
pUDE672	2 µm ori, *URA3*, p*TDH3*-*eGFP*-t*CYC1*	This study
pUDE1119	2 µm ori, *URA3*, p*TDH3*-*eGFP*19aa-t*CYC1*	This study
pUDE1120	2 µm ori, *URA3*, p*TDH3*-*eGFP*-*CLN2*_PEST_-t*CYC1*	This study

*Kl* *denotes* *Kluyveromyces lactis*

A unique gRNA sequence targeting p*DAN1*-*prk*-t*PGK1* was designed as described in BoxS4 in the supplementary materials in the publication by Mans et al. [60]. To construct plasmid pUDR760, which expresses the unique *prk*-targeting gRNA, the pROS10 backbone was PCR amplified with primer 5793 (double binding) and the plasmid insert was amplified using primer 17610 (double binding) and pROS10 as template for both reactions [61], after which pUDR760 was assembled by Gibson Assembly. A DNA repair fragment for eliminating a *prk* expression cassette, while simultaneously restoring the *SGA1* target site into which it had been integrated, was PCR amplified from genomic DNA of *S. cerevisiae* IMX581 with primers 11404 and 17612.

*DAN1* and *ANB1* promoter fragments were PCR amplified from genomic DNA of strain IMX581 with primer pairs 7978 and 7931 and 17929 and 17930, respectively. Genomic DNA of strain IMX581 was also used as template for amplification of the 534 3’-terminal nucleotides of *CLN2*, which contain the *CLN2*_*PEST*_ sequence [24]. Primer pairs 18391 and 17628 used in this amplification were designed to add flanking sequences with homology to *prk* and t*PGK1*, respectively. A DNA fragment that resulted in frame-shift of the *CLN2*_*PEST*_ sequence added to the *prk* ORF, was inadvertently obtained with primers 17627 and 17628. The resulting C-terminal extension of the PRK protein and will be referred to as 19aa tag. The *prk* ORF was amplified from pUDE046 with primer pair 7932 and 17626 to add flanks with homology to p*DAN1* and 19aa tag, with 7932 and 18392 to add flanks homologous to p*DAN1* and *CLN2*_*PEST*_, with 7932 and 7081 for adding flanks homologous to p*DAN1* and t*PGK1* and with 17983 and 7081 to add flanks with homology to p*ANB1* and t*PGK1*. A *PGK1* terminator fragment was amplified from genomic DNA of strain IMX581 with primer pair 7084 and 11205 or 17629 and 11205 to add flanks homologous to *prk* and to *CLN2*_*PEST*_ and 19aa tag, respectively. Complete expression cassettes of p*DAN1-prk-tPGK1,* p*DAN1*-*prk*-*CLN2*_*PEST*_-t*PGK1*, p*DAN1*-*prk*-19aa-t*PGK1* and p*ANB1-prk-tPGK1* were assembled by in vivo homologous recombination [62].

A linearized backbone of p426-*GPD* [63] was amplified with primers 10546 and 10547. The eGFP insert was ordered from GeneArt and amplified with primers 11584 and 11585. The insert was assembled with the p426-*GPD* fragment to obtain pUDE672. For construction of pUDE682, three fragments were amplified with primers 15651 and 18945, 16503 and 18946 and 5975 and 15645 with pUDE672 as template and one fragment using primers 18943 and 18944 and genomic DNA of IMX581 as template. Gibson assembly of the four fragments yielded pUD682, which carries a p*GPD*-eGFP-*CLN2*_pest_-t*CYC1* cassette. To obtain pUDE681, which encodes a p*GPD*-eGFP-19aa-t*CYC1* cassette, pUDE672 was amplified with primers 5975 and 15645, 18948 and 15651 and 18947 and 16503.

### Yeast strain construction

Yeast strains were transformed with lithium-acetate method [64] and transformation mixtures were plated on SMD plates. Correct Cas9-mediated integration [61] and/or assembly of DNA fragments was verified by diagnostic PCR. gRNA-expression plasmids were removed by overnight growth in liquid, non-selective YPD and subsequent isolation of single colonies on YPD plates. Plasmid loss was checked by streaking colonies on selective (SMD) and non-selective (YPD) plates. A single verified colony, restreaked thrice, was grown on non-selective liquid medium and stored at − 80 °C (Table 5).

**Table 5:** *S. cerevisiae* strains used in this study

Strain name	Relevant genotype	Parental strain	Origin
CEN.PK113-7D	*MATa URA3*	–	
CEN.PK113-5D	*MATa ura3-52*	–	
IMX581	*MATa ura3-52 can1::cas9-natNT2*	CEN.PK113-5D	[61]
IMX1489	*MATa ura3-52 can1::cas9-natNT2 gpd2::*non-ox PPP↑ *sga1::pDAN1-prk, cbbm (15 copies),* *groES*, *groEL* pUDR103 (*KlURA3*)	IMX581	[13, 23]
IMX2288	*MATa ura3-52 can1::cas9-natNT2 gpd2::*non-ox PPP↑ *sga1::*p*DAN1-prk, cbbm (2 copies), groES*, *groEL*	IMX1489	This study
IMX2545	*MATa ura3-52 can1::cas9-natNT2 gpd2::*non-ox PPP↑ *sga1::sga1*-gRNA site, *cbbm* (2 copies), *groES*, *groEL*	IMX2288	This study
IMX2593	*MATa ura3-52 can1::cas9-natNT2 gpd2::*non-ox PPP↑ *sga1::*p*DAN1-prk-*19aa*, cbbm* (2 copies), *groES*, *groEL* pUDR103 (*KlURA3*)	IMX2545	This study
IMX2608	*MATa ura3-52 can1::cas9-natNT2 gpd2::*non-ox PPP↑ *sga1::*p*ANB1-prk, cbbm* (2 copies), *groES*, *groEL* pUDR103 (*KlURA3*)	IMX2545	This study
IMX2701	*MATa ura3-52 can1::cas9-natNT2 gpd2::*non-ox PPP↑ *sga1::*p*DAN1-prk-CLN2*_*PEST*_*, cbbm* (2 copies), *groES*, *groEL* pUDR103 (*KlURA3*)	IMX2545	This study
IMX2736	*MATa ura3-52 can1::cas9-natNT2 gpd2::*non-ox PPP↑ *sga1::*p*DAN1-prk, cbbm* (2 copies), *groES*, *groEL* pUDR103 (*KlURA3*)	IMX2545	This study
IME678	*MATa ura3-52 can1::cas9-natNT2*, pUDE672 (*URA3*)	IMX581	This study
IME681	*MATa ura3-52 can1::cas9-natNT2*, pUDE1119 (*URA3*)	IMX581	This study
IME682	*MATa ura3-52 can1::cas9-natNT2*, pUDE1120 (*URA3*)	IMX581	This study

Non-ox PPP↑ indicates the integration of the expression cassettes of p*TDH3-RPE1*, p*PGK1-TKL1*, p*TEF1-TAL1*, p*PGI1-NQM1*, p*TPI1-RKI1* and p*PYK1-TKL2. Kl* denotes* Kluyveromyces lactis*

Strain IMX2288 was obtained by removing pUDR103 from *S. cerevisiae* IMX1489 (Δ*gpd2* p*DAN1*-*prk cbbm* (15x) pUDR103). After sequencing it was verified that strain IMX2288 and all strains made from this strain contained 2 copies of *cbbm* while IMX1489 contained 15 copies of *in tandem* integrated *cbbm*. Co-transformation of strain IMX2288 with pUDR760 (targeting *prk*) and the repair fragment (encoding the *sga1* target sequence) yielded strain IMX2545 (Δ*gpd2 cbbm* (2x)), which was used for construction of multiple congenic strains with different *prk*-cassettes. Co-transformation of strain IMX2545 with pUDR103 together with p*DAN1* sequence, *prk*-ORF, 19aa-tag sequence and the t*PGK1* fragment, yielded strain IMX2593 (Δ*gpd2* p*DAN1*-*prk*-19aa *cbbm* (2x) pUDR103). To construct strain IMX2608 (Δ*gpd2* p*ANB1*-*prk cbbm* (2x) pUDR103), strain IMX2545 was transformed with pUDR103 together with the promoter sequence of *ANB1*, *prk*-ORF and t*PGK1*. Strain IMX2701 (Δ*gpd2* p*DAN1*-*prk*-*CLN2*_*PEST*_* cbbm* (2x) pUDR103) was obtained by transformation of strain IMX2545 with pUDR103 and p*DAN1*, *prk*-ORF, *CLN2*_PEST_ and t*PGK1*. Transformation of strain IMX2545 with pUDR103 together with the promoter sequence of *DAN1*, *prk*-ORF and t*PGK1* resulted in strain IMX2736 (Δ*gpd2* p*DAN1*-*prk cbbm* (2x) pUDR103).

Strains IME678, IME681 and IME682 were obtained by transforming strain IMX581 with pUDE672, pUDE1119 and pUDE1120, respectively.

### Shake-flask cultivation

Aerobic shake-flask cultures of *S. cerevisiae* were grown at 30 °C in 500-mL round-bottom flasks containing 100 mL medium, placed in an Innova incubator (Eppendorf Nederland B.V., Nijmegen, The Netherlands) and shaken at 200 rpm. Anaerobic shake-flask cultures were grown in 50-mL round-bottom flasks containing 30 mL medium, incubated at 30 °C in a Bactron anaerobic chamber (Sheldon Manufacturing Inc., Cornelius, OR) under an atmosphere of 5% (v/v) H_2_, 6% (v/v) CO_2_ and 89% (v/v) N_2_, on a IKA KS 260 basic shaker (Dijkstra Verenigde BV, Lelystad, The Netherlands) at 200 rpm [65].

### Bioreactor cultivation

Anaerobic batch and chemostat cultures of *S. cerevisiae* strains were grown in 2-L bioreactors with a 1-L working volume (Applikon, Delft, The Netherlands), at 30 °C and at a stirrer speed of 800 rpm. Culture pH was maintained at 5.0 by automatic addition of 2 M KOH. Loss of water by evaporation was minimized by cooling of the off-gas to 4 °C in a condenser. Bioreactors were sparged with a 90:10 mixture of N_2_ and CO_2_ at a rate of 0.5 L min^−1^. Cultures were grown on SM supplemented with glucose, at concentrations of 20 g L^−1^ for batch cultures, 25 g L^−1^ for chemostat cultures and 59 g L^−1^ for nitrogen-limited cultures (on modified SM, see above). Antifoam C (Sigma Aldrich) was added to bioreactor media at a concentration of 0.2 g L^−1^. Oxygen diffusion into the bioreactors was minimized by the use of Norprene tubing and Viton O-rings [65]. Frozen stock cultures (1-mL samples) were used to inoculate aerobic shake-flasks on SMD. After incubation for 8–12 h, these initial cultures were used to inoculate shake-flask pre-cultures, which, upon reaching an OD_660_ between 3 and 6, were used to inoculate bioreactor cultures at an initial OD_660_ of approximately 0.2.

### Acetaldehyde determination

Analysis of acetaldehyde concentrations was based on a previously described protocol [18]. A solution of 0.2 M 4-dinitrophenylhydrazine in phosphoric acid (DNPH; Sigma-Aldrich) was diluted in acetonitrile (VWR International, Louvain, Belgium) to obtain a solution of 0.9 g L^−1^ DNPH in acetonitrile. Sampling tubes containing 5.00 mL (exact volume determined by weighing) of the resulting derivatization solution were stored at − 20 °C and placed in a cryostat at − 40 °C at least one hour before sampling. For analyses of acetaldehyde concentration in culture liquid, 1 mL of complete broth (including biomass) was sampled directly into 5.00 mL of derivatization solution using a rapid-sampling set-up [66]. After mixing on a Vortex Genie II (Scientific Industries inc., Bohemia, NY), the samples were incubated on a Nutating Mixer (VWR International) at 4 °C for 1–3 h. The weight was determined before and after sampling, to be able to determine the dilution factor. After centrifugation, the supernatant was stored at − 80 °C until analysis by HPLC. To measure acetaldehyde in bioreactor off-gas, the off-gas stream of a bioreactor was led through two bottles connected in series, each containing approximately 400 mL derivatization solution. The time period over which acetaldehyde was trapped and the exact volumes of the derivatization before and after were determined to enable corrections for evaporation. Samples from the two bottles were directly analysed by HPLC. Saturation of the derivatization solution in the first bottle was assessed by measuring the acetaldehyde-2,4-dinitrophenylhydrazine adduct (A-DNPH) in the second bottle. HPLC analysis was performed on a Waters WAT086344 silica-based reverse-phase C18 column operated at room temperature with a hydrophobic stationary phase, at a flow rate of 1 mL min^−1^. The mobile phase was made increasingly hydrophobic by linearly changing its composition from 100% MilliQ water to 100% acetonitrile (VWR international) over a period of 20 min. A-DNPH (Sigma-Aldrich) was dissolved in acetonitrile to a standard solution of 50.9 mg L^−1^, from which a calibration curve was prepared.

### Analytical methods

Biomass dry weight measurements, analyses of metabolite concentrations and correction for ethanol evaporation from bioreactor cultures were performed as described previously [67]. Metabolite concentrations in steady-state chemostat cultures were analysed after rapid quenching of the culture samples using cold steel beads [68]. High concentration of CO_2_ in the inlet gas hindered the accurate determination of carbon balances [12, 13]. Therefore, balances of the degree of reduction of substrates and products were used to calculate electron recoveries [21]. For organic compounds that only contain carbon, hydrogen and/or oxygen, degree of reduction (γ) represents the number of electrons released upon complete oxidation to CO_2_, H_2_O and or H^+^. These oxidized compounds are assigned a γ of zero, which yields values of γ for H, C and O of 1, 4 and 2, respectively, and for positive and negative charge of − 1 and 1, respectively. To simplify construction of degree-of-reduction balances, the nitrogen source was assigned γ = 0 which, with NH_4_^+^ as nitrogen source, implied that γ = 3 for N. Calculations were based on an estimated composition and degree of reduction of yeast biomass [69].

### Transcriptome analysis

For transcriptome analysis, culture samples containing 100 to 160 mg of cells were sampled directly into liquid nitrogen to prevent mRNA turnover [70]. Within 2 weeks after sampling, samples were transferred to a mixture of phenol/chloroform and TEA buffer for long-term storage at − 80 °C [71]. Total RNA extraction was performed using a phenolic acid/chloroform method as described previously [72]. Quality of the RNA was assessed with a NanoDrop 2000 spectrophotometer (Thermo Fisher Scientific) and RNA quality was determined by RNA screen tape using a Tapestation (Agilent technologies, Santa Clara, CA). RNA concentration was determined with a Qubit (Thermo Fisher Scientific). Paired-end sequencing was performed on a Truseq Stranded mRNA 150-bp insert library using Illumina SBS technology (Macrogen, Amsterdam, The Netherlands). Sequencing reads were aligned to the CEN.PK113-7D genome using STAR [73]. The expression was quantified by featureCounts (version 1.6.0) [74] and normalized to FPKM counts by applying the rpkm function from the edgeR package [75].

### Flow cytometry

GFP fluorescence was detected by flow cytometry. After growth on SMD to an OD_660_ of between 2 and 4 in aerobic shake-flask cultures, cycloheximide (25 mg L^−1^) was added to arrest protein synthesis and cells were suspended to ~ 1.0^.^ 10^6^ cells per mL of Isoton II Diluent (Beckman Coulter, Woerden, The Netherlands). At least 10,000 cells were analysed for green fluorescence (FL-1, 530/30 nm BP filter) after excitation by a 488 nm laser on a BD-Accuri C6 flow cytometer (Becton Dickinson, Franklin Lakes, New Jersey). Expression in *S. cerevisiae* of genes encoding fluorescent proteins from episomal 2-micron-based vectors has been shown to lead to a bimodal distribution of fluorescence, with a sizable non-fluorescent subpopulations [76, 77]. Cells of the reference strain *S. cerevisiae* IME324 were therefore used to optimize gating of non-fluorescent cells in experiments with the GFP-expressing strains IME678, IME681 and IME682. The raw data and applied gating can be found in the supplementary materials.

### Protein extraction and proteome analysis

For proteome analysis, 5 mg (dry weight) of cells were harvested from late-exponential-phase (OD_660_ between 8 and 10) anaerobic bioreactor batch cultures on SMD (20 g L^−1^ glucose), or from steady-state anaerobic chemostat cultures grown a at 0.05 h^−1^, 0.10 h^−1^ or 0.25 h^−1^. Cells were washed once and then resuspended in 5.00 mL ice-cold 1% NaCl. After storage at − 80 °C, the frozen samples were processed as described previously [13]. All LC–MS/MS results were searched against the *S. cerevisiae* protein database (UniProt), to which the amino acid sequences of the heterologous enzymes (PRK, CbbM) were manually added.

### Genome sequencing

Genomic DNA was isolated from yeast cultures grown on YPD medium with a blood & cell culture DNA kit with 100/G genomics-tips (Qiagen, Hilden, Germany) according to supplier’s instructions. Short-read whole-genome sequencing was performed in-house or commercially (Macrogen, Amsterdam; GenomeScan, Leiden) on a 350-bp insert TruSeq PCR-free library using the MiSeq platform (Illumina, San Diego, CA). Genomic libraries were either prepared in-house with a Nextera DNA Flex Library Prep kit (Illumina, San Diego, CA) and sequenced using the MiSeq platform (Illumina, San Diego, CA), or a 350-bp insert TruSeq PCR-free library was prepared and sequenced commercially using Illumina SBS technology (Macrogen; GenomeScan, Leiden, The Netherlands). Sequence reads were mapped against the CEN.PK113-7D genome, supplemented with an additional virtual contig encompassing the sequences of chromosomally integrated expression cassettes for non-native genes (p*DAN1*-*prk*-t*CPS1*, p*ANB1*-*prk*-t*CPS1*, p*DAN1-prk-CLN2*_*PEST*_-t*CPS1*, p*DAN1*-*prk*-19aa-t*CPS1* p*TDH3*-*cbbm*-t*CYC1*, p*TEF1*-*groEL*-t*ACT1*, and p*TPI1*-*groES*-tPGI1) and processed as described previously [22]. The copy number of *cbbm* was estimated through comparison of the read depth of *cbbm* to the average read depth for all chromosomes.

Long-read sequencing was performed in-house using the MinION platform using R10.3 flow cells, for 48 h (Oxford Nanopore Technologies, Oxford, UK). Genomic libraries were prepared using Oxford Nanopore Sequencing kit SQK-LSK109, according to manufacturer’s instructions. Basecalling was performed using Guppy version 4.5.4 (Oxford Nanopore Technologies), ran with options –flowcell FLO-MIN111 and –kit SQK-LSK109. Resulting basecalled files were demultiplexed by applying Guppy_barcoder (Oxford Nanopore Technologies) with option–barcode_kits EXP-NBD104. The demultiplexed samples were assembled using canu version 2.0 [78].

## Supplementary Information

**Additional file 1**: Measurement data of anaerobic glucose-limited steady-state chemostat cultures of IME324 and IMX1489 at 0.05, 0.1 and 0.25 h^−1^. Data were used to prepare Figure 2 and Table 1.

**Additional file 2**: The FPKM normalized counts of the annotated unique open reading frames in CEN.PK113-7D for anaerobic glucose-limited steady-state chemostat cultures of IME324 and IMX1489 at 0.05, 0.1 and 0.25 h^−1^ and anaerobic nitrogen-limited steady-state chemostat cultures of IME324 and IMX1489 at 0.1 h^−1^. Data were used to prepare Figure 5.

**Additional file 3**: Measurement data of anaerobic batch cultures of IME324, IMX1489, IMX2736, IMX2701, IMX2593 and IMX2608 on 20 g L^−1^ of glucose. Data were used to prepare Table 2 and Figure 4.

**Additional file 4**: Measurement data of anaerobic glucose-limited steady-state chemostat cultures of IME324, IMX1489, IMX2736, IMX2701, IMX2593 and IMX2608 at 0.05 h^−1^. Data were used to prepare Table 3 and Figure 3A.

**Additional file 5**: Peptide abundance determined by LC-MS of anaerobic steady-state chemostat cultures and anaerobic mid-exponential phase batch cultures of PRK/RuBisCO expressing strains IMX1489, IMX2736, IMX2701, IMX2593 and IMX2608. Measurement data were used to prepare Figure 3B.

**Additional file 6**: Table S6: Oligonucleotides used in this study. Table S2: DNA and protein sequences of *CLN2*_*PEST*_ and inadvertently obtained 19aa tag. Figure S1: glycerol, acetate and acetaldehyde production in anaerobic nitrogen limited *S. cerevisiae* chemostat cultures of IME324 and IMX1489 at a dilution rate of 0.1 h^−1^. Figure S2: fluorescence density plot of *S. cerevisiae* strains IME678, IME681and IME682, measured over time after the addition of cycloheximide. Table S3: Frequency of the triplets encoding the 19-amino-acid C-terminal extension of PRK in the yeast genome. Figure S3: Growth, glucose consumption, ethanol formation, acetaldehyde formation and glycerol formation in anaerobic bioreactor batch cultures of *S. cerevisiae* strains IME324, IMX1489, IMX2736, IMX2701, IMX2593 and IMX2608.

**Additional file 7**: Overview of the raw flow cytometry data and the gating strategy applied. Supporting data for figure S2.

## Data Availability

Short read DNA sequencing data of the *Saccharomyces cerevisiae* strains IMX2288, IMX2593, IMX2701 and IMX2736 and long read DNA sequencing data of the *Saccharomyces cerevisiae* strains IMX1489 and IMX2608 were deposited at NCBI under BioProject accession number PRJNA906461. Transcriptomics datasets of IMX1489 and IME324 of anaerobic steady state C-limited chemostat cultures at 0.05 h^−1^, 0.1 h^−1^ and 0.25 h^−1^ and N-limited chemostat cultures at 0.1 h^−1^ were uploaded to GEO under accession number GSE221392. All measurement data used to prepare Figs. 2, 3, 4 and 5, Tables 1, 2 and 3 of the manuscript are available in Additional files 1, 2, 3, 4 and 5.
