# Supplementary material for: Quantification and mitigation of byproduct formation by low-glycerol-producing Saccharomyces cerevisiae strains containing Calvin-cycle enzymes
Source: Biotechnol Biofuels Bioprod. 2023 May 12;16:81. doi: 10.1186/s13068-023-02329-9 (PMC10176687; doi:10.1186/s13068-023-02329-9)
Supplement: Supplementary file 6 — Additional file 6: Table S6: Oligonucleotides used in this study. Table S2: DNA and protein sequences of CLN2PEST and inadvertently obtained 19aa tag. Figure S1: glycerol, acetate and acetaldehyde production in anaerobic nitrogen limited S. cerevisiae chemostat cultures of IME324 and IMX1489 at a dilution rate of 0.1 h−1. Figure S2: fluorescence density plot of S. cerevisiae strains IME678, IME681and IME682, measured over time after the addition of cycloheximide. Table S3: Frequency of the triplets encoding the 19-amino-acid C-terminal extension of PRK in the yeast genome. Figure S3: Growth, glucose consumption, ethanol formation, acetaldehyde formation and glycerol formation in anaerobic bioreactor batch cultures of S. cerevisiae strains IME324, IMX1489, IMX2736, IMX2701, IMX2593 and IMX2608. [file 13068_2023_2329_MOESM6_ESM.docx]

**Additional files**

**Table S1: Oligonucleotides used in this study.** HPLC refers to High Pressure Liquid Chromatography; PAGE denotes PolyAcrylamide Gel Electrophoresis; DST stands for desalted.

| **name** | **Sequence 5’ 🡪 3’** | **Purification method** |
| --- | --- | --- |
| 5975 | AACGAGCTACTAAAATATTGCGAA | HPLC |
| 7081 | ATTGATCTATCGATTTCAATTCAATTCAATCTAGGCTTTAGCAGCTGTTG | PAGE |
| 7084 | ATTGAATTGAATTGAAATCGATAG | PAGE |
| 7931 | TACTTGGGGTATATATTTAGTATGC | PAGE |
| 7932 | TTGCAGATAAAAGTGTAGCAGATAAAAGTGTAGCATACTAAATATATACCCCAAGTAATGTCACAACAACAAACAATTGTG | PAGE |
| 7978 | GCATAGAACATTATCCGCGGAAACGGGTATTAGGGGTGAGGGTGAATAAGGAAAGTCAGGGAAATCGGGCAGAATGCAAGGGCAAACAGG | DST |
| 10546 | ATCCGTCGAAACTAAGTTCTG | PAGE |
| 10547 | TCATGTAATTAGTTATGTCACGC | PAGE |
| 11205 | TGCCGAACTTTCCCTGTATGAAGCGATCTGACCAATCCTTTGCCGTAGTTTCAACGTATGGCTTCAAGCTTACACAACAC | DST |
| 11404 | TTATCCGCGGAAACGGGTATTAGGGGTGAGGGTGAATAAGGAAAGTCAGGGAAATCGGGC | DST |
| 11584 | TTTTTTAGTTTTAAAACACCAGAACTTAGTTTCGACGGATATGGTGAGCAAGGGCGAGGAG | PAGE |
| 11585 | GGAGGGCGTGAATGTAAGCGTGACATAACTAATTACATGATTACTTGTACAGCTCGTCCATGCC | PAGE |
| 15645 | TGAAGTGGTGGCCTAACTACGG | DST |
| 15651 | AATATTTTAGTAGCTCGTTACAGTCCG | DST |
| 16503 | TTCTGCGCGTAATCTGCTGC | DST |
| 17610 | TGCGCATGTTTCGGCGTTCGAAACTTCTCCGCAGTGAAAGATAAATGATCTATTCAACCTCCTAAGATCTGTTTTAGAGCTAGAAATAGCAAGTTAAAATAAGGCTAGTCCGTTATCAAC | PAGE |
| 17612 | AGGCTTCGCTAATCTGCGCGGCCCGAACTTTCCCTGTATGAAGCGATCTGACCAATCCTTTACGACAGCGACTTGTCCACTGCAGACAGT | DST |
| 17626 | TTCGAAAGAGCATGATGGGGTTGATATGGTAAGCTTTCTCGAAATGTTCGGCTTTAGCAGCTGTTGCAGTTGCTGTAGAC | DST |
| 17627 | GAGCAGCTCGTTGCTAGCAGGTCTACAGCAACTGCAACAGCTGCTAAAGCCGAACATTTCGAGAAAGCTTACCATATCAA | DST |
| 17628 | AGAAAAGAAAAAAATTGATCTATCGATTTCAATTCAATTCAATCTAATTACTTGGGTATTGCCCATACCAAAAGAAATTAA | DST |
| 17629 | GGTATGGGCAATACCCAAGTAATTAGATTGAATTGAATTGAAATCGATAGATCAATTTTTTTCTTTTCTCTTTCCCCATC | DST |
| 17929 | TTATCCGCGGAAACGGGTATTAGGGGTGAGGGTGAATAAGGAAAGTCAGGGAAATCGGGCAAAAGTGTAGCACCTTTCTTAGCAGAACCGGCCTTGAATT | DST |
| 17930 | CAACCTGAATCTGCTGCTAAACCAATCACAATTGTTTGTTGTTGTGACATGTTTTAGTGTGTGAATGAAATAGGTGTATGTTTTCTTTTTGCTAGACAATA | DST |
| 17983 | ATTGTCTAGCAAAAAGAAAACATACACCTATTTCATTCACACACTAAAACATGTCACAACAACAAACAATTGTGATTGGTTTAGCAGCAGA | DST |
| 18391 | CGAGCAGCTCGTTGCTAGCAGGTCTACAGCAACTGCAACAGCTGCTAAAGCCGCATCCAACTTGAACATTTCGAGAAAGCTTACCATA | DST |
| 18392 | GCATGATGGGGTTGATATGGTAAGCTTTCTCGAAATGTTCAAGTTGGATGCGGCTTTAGCAGCTGTTGCAGTTGCTGTAGACCTGCTA | DST |
| 18943 | GCTGGAGTTCGTGACCGCCGCCGGGATCACTCTCGGCATGGACGAGCTGTACAAGGCATCCAACTTGAACATTTCGAGAAAGCTTACCATATCAAC | DST |
| 18944 | GCGGATGTGGGGGGAGGGCGTGAATGTAAGCGTGACATAACTAATTACATGATTAAATTACTTGGGTATTGCCCATACCAAAAGAAATTAAAGATG | DST |
| 18945 | GCCTCATCTTTAATTTCTTTTGGTATGGGCAATACCCAAGTAATTTAATCATGTAATTAGTTATGTCACGCTTACATTCACGC | DST |
| 18946 | CATGATGGGGTTGATATGGTAAGCTTTCTCGAAATGTTCAAGTTGGATGCCTTGTACAGCTCGTCCATGCCGAGAGTGAT | DST |
| 18947 | TTATTTGAATTTTCGAAAGAGCATGATGGGGTTGATATGGTAAGCTTTCTCGAAATGTTCCTTGTACAGCTCGTCCATGCCGAGAGTGAT | DST |
| 18948 | GAACATTTCGAGAAAGCTTACCATATCAACCCCATCATGCTCTTTCGAAAATTCAAATAATCATGTAATTAGTTATGTCACGCTTACATTCACGC | DST |

**Table S2** DNA sequences and predicted amino-acid sequences of *CLN2_PEST_* and frameshifted *CLN2_PEST_* (19aa tag).The DNA sequence of the serendipity tag is underlined in both DNA sequences.

|  |  | **Sequence 5’🡪 3’** |
| --- | --- | --- |
| ***CLN2_PEST_*** | DNA sequence | GCATCCAACTTGAACATTTCGAGAAAGCTTACCATATCAACCCCATCATGCTCTTTCGAAAATTCAAATAGCACATCCATTCCTTCGCCCGCTTCCTCATCTCAAAGCCACACTCCAATGAGAAACATGAGCTCACTCTCTGATAACAGCGTTTTCAGCCGGAATATGGAACAATCATCACCAATCACTCCAAGTATGTACCAATTTGGTCAGCAGCAGTCAAACAGTATATGTGGTAGCACCGTTAGTGTGAATAGTCTGGTGAATACAAATAACAAACAAAGGATCTACGAACAAATCACGGGTCCTAACAGCAATAACGCAACCAATGATTATATTGATTTGCTAAACCTAAATGAGTCTAACAAGGAAAACCAAAATCCCGCAACGGCGCATTACCTCAATGGGGGCCCACCCAAGACAAGCTTCATTAACCATGGAATGTTCCCCTCGCCAACTGGGACCATAAATAGCGGTAAATCTAGCAGTGCCTCATCTTTAATTTCTTTTGGTATGGGCAATACCCAAGTAATATAG |
|  | Protein sequence | ASNLNISRKLTISTPSCSFENSNSTSIPSPASSSQSHTPMRNMSSLSDNSVFSRNMEQSSPITPSMYQFGQQQSNSICGSTVSVNSLVNTNNKQRIYEQITGPNSNNATNDYIDLLNLNESNKENQNPATAHYLNGGPPKTSFINHGMFPSPTGTINSGKSSSASSLISFGMGNTQVI |
| **19aa tag** | DNA sequence | GAACATTTCGAGAAAGCTTACCATATCAACCCCATCATGCTCTTTCGAAAATTCAAATAG |
|  | Protein sequence | EHFEKAYHINPIMLFRKFK |

**Table S3** Frequency of the triplets encoding the 19-amino-acid C-terminal extension of PRK in the yeast genome. Triplets whose frequency of occurrence in the *S. cerevisiae* genome is below half of the frequency of occurrence of the most abundant synonymous triplet are indicated in bold. Information on triplet frequency in the *S. cerevisiae* genome was derived from https://www.genscript.com/tools/codon-frequency-table.

| **Triplet** | **Amino acid** | **Frequency**  **of triplet**  **‰** | **Number of triplets**  **for amino acid** | **Frequency**  **rank** | **Frequency**  **synonymous triplet(s)**  **‰** | **% of frequency**  **most abundant**  **synonomous triplet** |
| --- | --- | --- | --- | --- | --- | --- |
| GAA | E | 45.9 | 2 | 1 | 19.1 | 100 |
| CAT | H | 13.7 | 2 | 1 | 7.8 | 100 |
| TTC | F | 18.2 | 2 | 2 | 26.1 | 69.7 |
| **GAG** | **E** | **19.1** | **2** | **2** | **45.9** | **41.6** |
| AAA | K | 42.2 | 2 | 1 | 30.8 | 100 |
| GCT | A | 21.1 | 4 | 1 | 16.2, 12.5, 6.1 | 100 |
| TAC | Y | 14.7 | 2 | 2 | 18.8 | 78.2 |
| CAT | H | 13.7 | 2 | 1 | 7.8 | 100 |
| ATC | I | 17.1 | 3 | 3 | 30.2, 17.8 | 56.6 |
| AAC | N | 24.9 | 2 | 2 | 36.0 | 69.1 |
| **CCC** | **P** | **6.8** | **4** | **3** | **18.2, 13.6, 5.2** | **37.4** |
| ATC | I | 17.1 | 3 | 3 | 30.2, 17.8 | 56.6 |
| ATG | M | 20.9 | 1 | 1 | - | 100 |
| **CTC** | **L** | **5.4** | **4** | **4** | **13.4, 12.2, 10.4** | **40.2** |
| TTT | F | 26.1 | 2 | 1 | 18.2 | 100 |
| **CGA** | **R** | **3.0** | **4** | **3** | **6.5, 2.6, 1.7** | **46.2** |
| AAA | K | 42.2 | 2 | 1 | 30.8 | 100 |
| TTC | F | 18.2 | 2 | 2 | 26.1 | 69.7 |
| AAA | K | 42.2 | 2 | 1 | 30.8 | 100 |

**Figure S1:** Yields of acetaldehyde and acetate on glucose and stoichiometric relationships between glycerol production and biomass formation in anaerobic nitrogen-limited and glucose-limited chemostat cultures of *S. cerevisiae* strains IME324 (reference strain lacking PRK-RuBisCO bypass) and IMX1489 (Δ*gpd2*, non-ox PPP↑, p*DAN1*-*prk*, 15x *cbbm*, *GroES/GroEL*), at a dilution rate of 0.1 h^-1^. Values represent means and individual values of measurements on independent steady-state duplicate cultures.

*
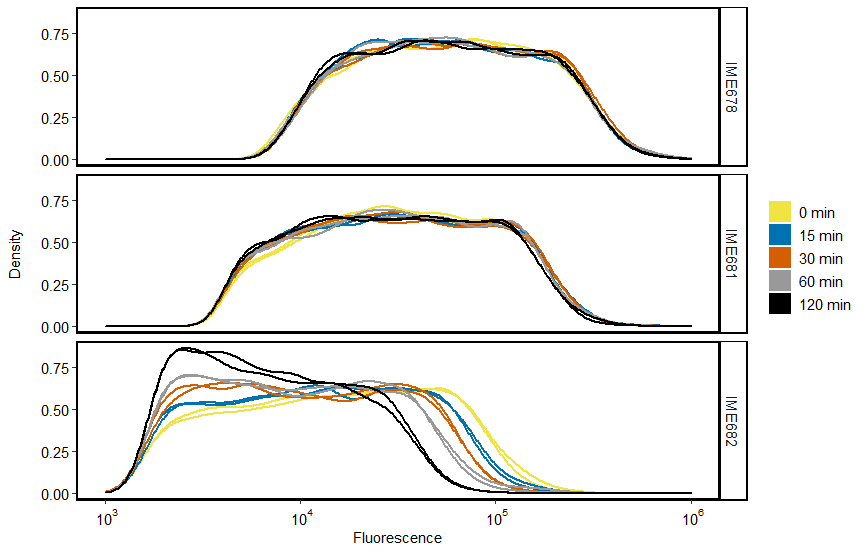
*

**Figure S2:** Fluorescence intensity plots of exponentially growing aerobic shakeflask cultures of S. cerevisiae strains IME678 (eGFP), IME681 (eGFP-19aa tag) and IME682 (eGFP-CLN2_PEST_) on SMD (pH=6), measured over time after the addition of cycloheximide. Data used in this plot were obtained by gating the raw flow-cytometry data as shown in Additional File 7.

**
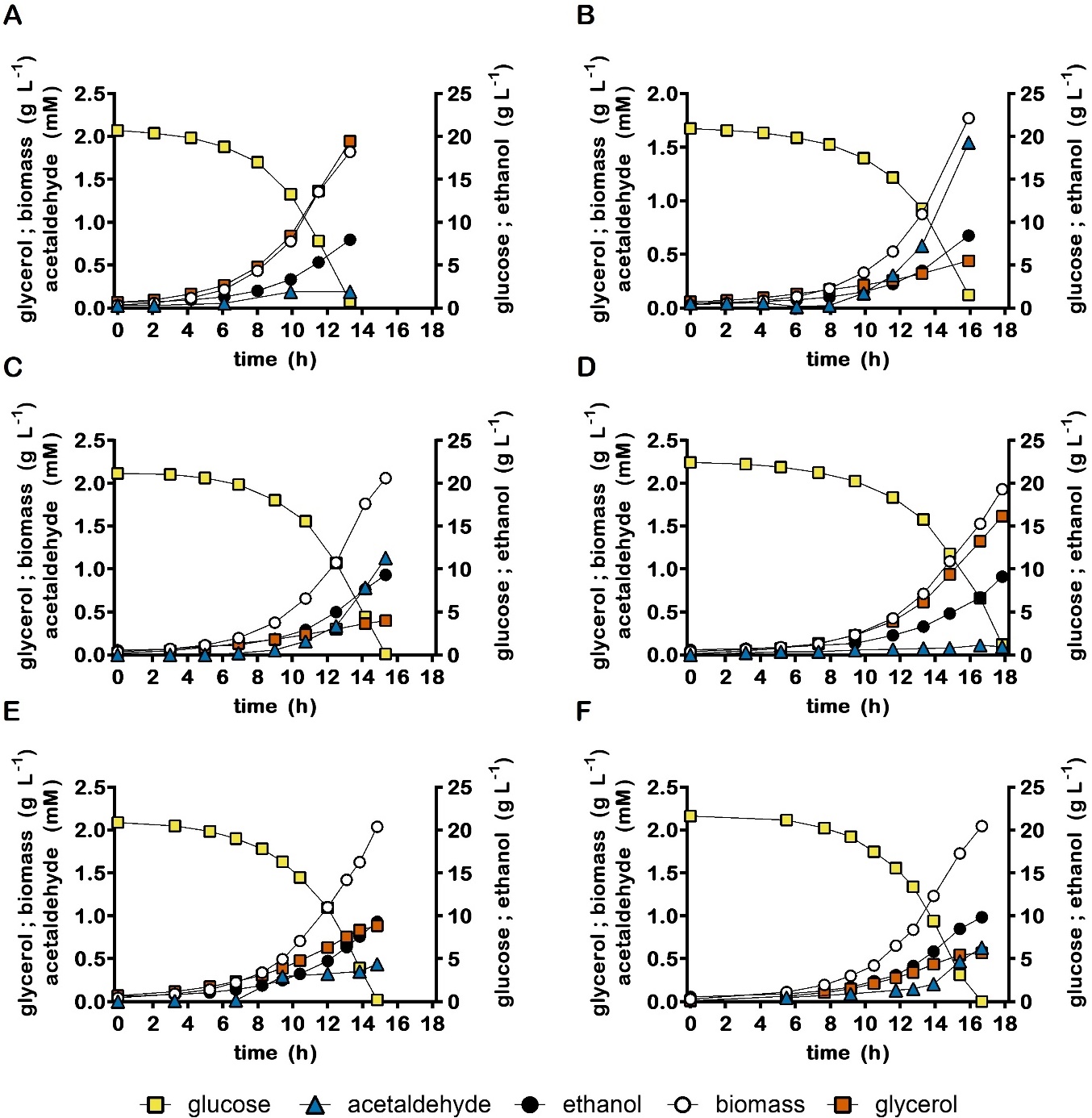
Figure S3:** Growth, glucose consumption, ethanol formation, acetaldehyde formation and glycerol formation in anaerobic bioreactor batch cultures of *S. cerevisiae* strains IME324 (reference strain lacking PRK/RuBisCO bypass) (**A**), IMX1489 (*Δgpd2*, non-ox PPP↑, p*DAN1*-*prk*, 15x *cbbm*, *GroES*/*GroEL*)(**B**), IMX2736 (*Δgpd2*, non-ox PPP↑, p*DAN1*-*prk*, 2x *cbbm*, *GroES*/*GroEL*)(**C**), IMX2701 (*Δgpd2*, non-ox PPP↑, p*DAN1*-*prk*-*CLN2_PEST_*, 2x *cbbm*, *GroES*/*GroEL*)(**D**), IMX2593 (*Δgpd2*, non-ox PPP↑, p*DAN1*-*prk*-19aa tag, 2x *cbbm*, *GroES*/*GroEL*)(**E**) and IMX2608 (*Δgpd2*, non-ox PPP↑, p*ANB1*-*prk*, 2x *cbbm*, *GroES*/*GroEL*)(**F**). Cultures were grown anaerobically at pH 5 and at 30 °C on synthetic medium containing 20 g L^-1^ glucose. Representative cultures of independent duplicate experiments are shown*,* corresponding replicate of each culture shown in Figure 3.
