# Supplementary material for: Quantification and mitigation of byproduct formation by low-glycerol-producing Saccharomyces cerevisiae strains containing Calvin-cycle enzymes
Source: Biotechnol Biofuels Bioprod. 2023 May 12;16:81. doi: 10.1186/s13068-023-02329-9 (PMC10176687; doi:10.1186/s13068-023-02329-9)
Supplement: Supplementary file 7 — Additional file 7: Overview of the raw flow cytometry data and the gating strategy applied. Supporting data for figure S2. [file 13068_2023_2329_MOESM7_ESM.docx]

**Overview raw data flow cytometry experiment (Figure S2)**

**Step 1 (left)** : SSC-A vs FSC-A plot was used to gate the cells

**Step 2 (right)** : SSC-A vs FL1-H plot was used to gate fluorescent population (using only the gated population from step 1)

**Raw data and applied gating for IME678**

-duplicate samples at t_0_

-duplicate samples at t_15_

-duplicate samples at t_30_

-duplicate samples at t_60_

-duplicate samples at t_120_

**Raw data and applied gating for IME681**

-duplicate samples at t_0_

-duplicate samples at t_15_

-duplicate samples at t_30_

-duplicate samples at t_60_

-duplicate samples at t_120_

**Raw data and applied gating for IME682**

-duplicate samples at t_0_

-duplicate samples at t_15_

-duplicate samples at t_30_

-duplicate samples at t_60_

-duplicate samples at t_120_
